# Kidney replacement therapy: trends in incidence, treatment, and outcomes of myocardial infarction and stroke in a nationwide Scottish study

**DOI:** 10.1093/eurheartj/ehae080

**Published:** 2024-03-01

**Authors:** Peter J Gallacher, David Yeung, Samira Bell, Anoop S V Shah, Nicholas L Mills, Neeraj Dhaun

**Affiliations:** BHF/University Centre for Cardiovascular Science, Little France Crescent, University of Edinburgh, Edinburgh EH16 4SA, UK; BHF/University Centre for Cardiovascular Science, Little France Crescent, University of Edinburgh, Edinburgh EH16 4SA, UK; Division of Population Health and Genomics, University of Dundee, Dundee, UK; Scottish Renal Registry, Scottish Health Audits, Public Health Scotland, Glasgow, UK; Department of Non-Communicable Epidemiology, London School of Hygiene and Tropical Medicine, London, UK; BHF/University Centre for Cardiovascular Science, Little France Crescent, University of Edinburgh, Edinburgh EH16 4SA, UK; Usher Institute, University of Edinburgh, Edinburgh, UK; BHF/University Centre for Cardiovascular Science, Little France Crescent, University of Edinburgh, Edinburgh EH16 4SA, UK; Department of Renal Medicine, Royal Infirmary of Edinburgh, Edinburgh, UK

**Keywords:** Myocardial infarction, Stroke, Kidney replacement therapy, Dialysis, Kidney transplant, Epidemiology

## Abstract

**Background and Aims:**

Patients with kidney failure have a higher risk of cardiovascular disease compared with the general population. Whilst temporal trends of myocardial infarction and stroke are declining in the general population, these have not been evaluated in patients with kidney failure. This study aimed to describe national trends in the incidence, treatment, and outcomes of myocardial infarction and stroke in patients with kidney failure (i.e. on dialysis or with a kidney transplant) over a 20-year period, stratified by age and sex.

**Methods:**

In this retrospective national data linkage study, all patients with kidney failure in Scotland (UK) receiving kidney replacement therapy between January 1996 and December 2016 were linked to national hospitalization, prescribing, and death records. The primary outcomes were the incidence of myocardial infarction and stroke, and subsequent cardiovascular death. Generalized additive models were constructed to estimate age-standardized, sex-stratified incidence rates and trends in cardiovascular and all-cause death.

**Results:**

Amongst 16 050 patients with kidney failure [52 (SD 15) years; 41.5% women], there were 1992 [66 (SD 12) years; 34.8% women] and 996 [65 (SD 13) years; 45.1% women] incident myocardial infarctions and strokes, respectively, between January 1996 and December 2016. During this period, the age-standardized incidence of myocardial infarction per 100 000 decreased in men {from 4376 [95% confidence interval (CI) 3998–4785] to 1835 (95% CI 1692–1988)} and women [from 3268 (95% CI 2982–3593) to 1369 (95% CI 1257–1491)]. Similarly, the age-standardized incidence of stroke per 100 000 also decreased in men [from 1978 (95% CI 1795–2175) to 799 (95% CI 729–875)] and women [from 2234 (95% CI 2031–2468) to 903 (95% CI 824–990)]. Compared with the general population, the incidence of myocardial infarction was four- to eight-fold higher in patients with kidney failure, whilst for stroke it was two- to four-fold higher. The use of evidence-based cardioprotective treatment increased over the study period, and the predicted probability of cardiovascular death within 1 year of myocardial infarction for a 66-year-old patient with kidney failure (mean age of the cohort) fell in men (76.6% to 38.6%) and women (76.8% to 38.8%), and also decreased in both sexes following stroke (men, from 63.5% to 41.4%; women, from 67.6% to 45.8%).

**Conclusions:**

The incidence of myocardial infarction and stroke has halved in patients with kidney failure over the past 20 years but remains significantly higher than in the general population. Despite improvements in treatment and outcomes, the prognosis of these patients following myocardial infarction and stroke remains poor.

## Structured Graphical Abstract

**Structured Graphical abstract:**
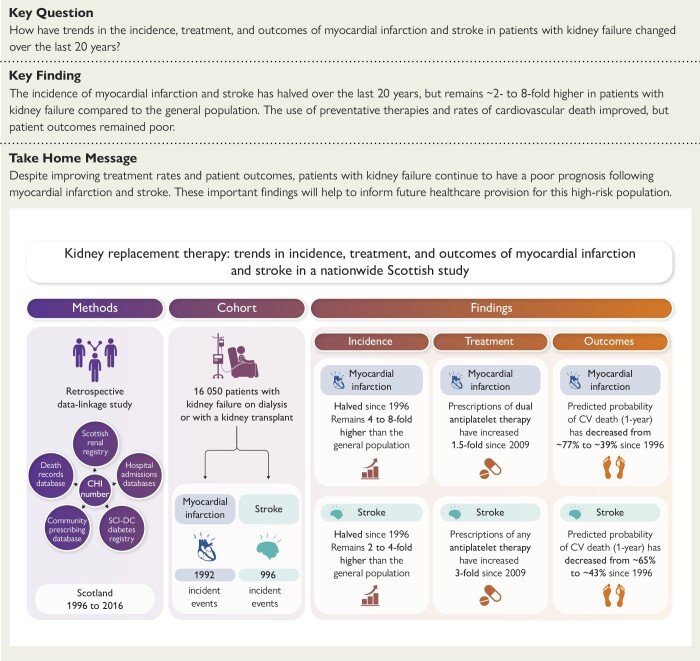
Schematic summarizing study methods, cohort, and key findings, including temporal trends in the incidence, treatment, and outcomes of myocardial infarction and stroke in patients with kidney failure in Scotland between 1996 and 2016. CHI, community health index; CV, cardiovascular.

**See the editorial comment for this article ‘Myocardial infarction and stroke in patients with kidney failure: can we do better?’, by M. Wyld and A.C. Webster, https://doi.org/10.1093/eurheartj/ehae153.**

## Introduction

Chronic kidney disease (CKD) is an increasingly important non-communicable disease which now affects 15%–20% of the world’s population.^1^ Cardiovascular disease is the most common complication of CKD.^2^ Cardiovascular risk increases as kidney function declines and is highest in patients with kidney failure.^3,4^ Globally, the number of patients living with kidney failure is ∼4–9 million, whilst the number of patients receiving kidney replacement therapy (KRT) (i.e. haemodialysis, peritoneal dialysis, or kidney transplantation) continues to rise as a consequence of improved patient survival.^5^ By 2030, it is expected that almost 5.5 million people with kidney failure will be receiving KRT worldwide, with the greatest increases observed in Asia.^6^

In the general population, there have been significant reductions in the incidence of myocardial infarction and stroke over the past 25 years,^7,8^ mostly as a consequence of improvements in the management of traditional cardiovascular risk factors, such as cigarette smoking, hypertension, and hypercholesterolaemia.^9^ Outcomes following myocardial infarction and stroke have also improved in the general population during this period,^9^ in part due to advances in the diagnosis and management of these conditions, including the introduction of more sensitive diagnostic tests and more effective therapies.^10,11^

Accurate and contemporaneous data describing temporal trends in myocardial infarction and stroke in patients with kidney failure are essential to provide an evidence base for public health policy and to inform planning of healthcare resource allocation for all patients with kidney disease. Previous studies have focused only on the incidence^12,13^ or outcomes^14,15^ of either myocardial infarction or stroke in patients with kidney failure, whilst very few have additionally reported national, individual patient-level prescribing data.^16^ Currently, it is unclear which cardiovascular treatments and interventions have been implemented into clinical practice, or how prescribing trends have changed over time in patients with kidney failure. In the coming years, these high-fidelity prescribing data will also provide an exciting opportunity to assess the real-world implementation and impact of recent, novel evidence-based therapies including sodium–glucose co-transporter-2 inhibitors, glucagon-like peptide 1 receptor agonists, and non-steroidal mineralocorticoid receptor antagonists on temporal trends of cardiovascular disease in patients with kidney failure.^17^ In this national, population-based data linkage study, we aimed to investigate temporal trends in the incidence, treatment, and outcomes of myocardial infarction and stroke in patients with kidney failure on dialysis or with a kidney transplant over the last 20 years.

## Methods

### Study design and data sources

We performed a national, population-based data linkage study utilizing multiple, routinely collected healthcare datasets comprising individual-level data from patients with kidney failure resident in Scotland, UK (see Supplementary*Text S1*; Supplementary data online, *Figure S1*). The NHS Public Health Scotland team performed the data linkage and provided access to a secure analytics platform within an ISO27001 and Scottish Government-accredited secure National Safe Haven for data cleaning and analysis.

### Study population

All patients with kidney failure aged ≥18 years old included in the Scottish Renal Registry (see description in Supplementary*Text S1*) and receiving KRT (i.e. haemodialysis, peritoneal dialysis, or kidney transplantation) for ≥90 days between 1 January 1996 and 31 December 2016 were eligible for inclusion. After linking these data with national hospital admission records, those patients with an incident episode (i.e. index event) of myocardial infarction or stroke occurring between 1 January 1996 and 31 December 2016—but after the initiation of KRT—were included in the final study cohort.

Incident non-fatal and fatal episodes of myocardial infarction and stroke were identified from national hospitalization and death records using International Classification of Diseases (ICD) 9 and 10 coding (see Supplementary data online, *Table S1*).^9^ Consistent with our prior studies,^18^ a 5-year lookback period was applied to exclude prior myocardial infarction or stroke and to ensure that the study population comprised incident events. To optimize specificity and sensitivity, we included only incident episodes with an ICD-9/-10 code for myocardial infarction or stroke appearing in the first two positions of the national hospital admission and death records.^18^ Data relating to the incidence of myocardial infarction and stroke in the general population were obtained following a direct request to the corresponding author of a recently published study;^9^ these data were used to calculate incidence rate ratios (IRRs) comparing patients with kidney failure with the general population.

### Population characteristics

#### Patient demographics

Patient age, sex, and social deprivation status were identified from national hospitalization records at the time of the incident episode. Social deprivation status was measured using the Scottish Index of Multiple Deprivation (SIMD), a validated measure of deprivation determined by social factors related to residential address (post code).^19^ Patients were assigned an SIMD quintile (1, most deprived; 5, least deprived) based on their individual SIMD rank at the time of their incident episode.

#### Primary kidney disease and KRT modality

Data relating to the cause of each patient’s kidney failure, the date and modality of first KRT, and KRT modality at the time of the incident episode were extracted from linked Scottish Renal Registry records. The primary kidney disease diagnosis groupings used in the study analyses are described on the Scottish Renal Registry website.^20^

#### Comorbidities and prescribed medications

Using linked national hospital admission records from the 5 years prior to each incident episode, patient comorbidities including prior myocardial infarction, stroke, heart failure, and coronary revascularization were identified using relevant ICD-9/-10 and Office of Population, Censuses and Survey’s Classification of Surgical Operations and Procedures (OPCS) codes (see Supplementary data online, *Table S1*) appearing in any position of the national hospital admission record. From 1 January 2009, incident episodes were additionally linked to individual patient-level prescribing data obtained from national community prescribing records. Anatomical Therapeutic Chemical (ATC) codes were used to identify dispensed prescriptions indicative of other underlying comorbidities, including chronic respiratory disease, diabetes mellitus, gastro-oesophageal reflux disease, and gout.^18^ ATC codes were also used to identify dispensed prescriptions in the year prior to and 6 months following incident episodes, including prescriptions of antiplatelet agents, angiotensin-converting enzyme (ACE) inhibitors, angiotensin receptor blockers (ARBs), beta-blockers, calcineurin inhibitors, mycophenolate mofetil, and statins (see Supplementary data online, *Table S1*).

### Follow-up and outcomes

Patients were followed-up until date of death or 31 December 2019, whichever was earliest. Causes of death were determined following the identification of relevant ICD-9/-10 codes in linked National Records of Scotland death records (see Supplementary*Text S1*; Supplementary data online, *Table S1*). The primary outcomes were the incidence of myocardial infarction and stroke, and cardiovascular death. Secondary outcomes included subsequent myocardial infarction or stroke, heart failure, bleeding, coronary revascularization, and all-cause death. The primary outcome of cardiovascular death was reported at 30 days, 1 and 3 years, whilst secondary outcomes were reported at 1 and 3 years.

### Missing data

Data relating to age, sex, comorbidities, and outcomes were complete for all patients with incident myocardial infarction and stroke, whilst prescribing data were complete for all patients from 2009 onwards. Missing data relating to social deprivation status and KRT modality were handled using complete case analysis.

### Statistical analysis

Baseline clinical characteristics, prescribed medications before and after incident episodes, and crude outcome data were presented according to calendar year and stratified by KRT modality at the time of the incident event. Continuous variables were presented as means and standard deviation (SD) or medians and interquartile ranges [IQRs] according to whether their distribution was normal or non-normal, respectively, whilst categorical variables were presented as percentages.

Incidence rates for myocardial infarction and stroke in patients with kidney failure were estimated according to age, sex, and KRT modality at the time of the event using generalized additive models, as previously described.^18,21^ To account for non-linearity in event year, regression models were fitted using non-parametric smooth terms (penalized thin-plate regression splines). In these models, a log-link and Poisson error distribution were specified, with a quasi-Poisson scaling factor to allow for overdispersion. Annual incidence rates were standardized against the European Standard Population.^22^ Annual IRRs were calculated for the period 1996–2014 using aggregate count and person-time data relating to the incidence of myocardial infarction and stroke in the Scottish general population,^9^ stratified by age, sex, and KRT modality. To evaluate cardiovascular death, generalized linear models with a logit-link and binomial error distribution were constructed. Following evaluation of model fit, these models were fitted with multivariable fractional polynomials to account for non-linearity in event year and patient age. These models included adjustment for age, sex, social deprivation status, and comorbidities (i.e. prior heart failure, myocardial infarction, or stroke).

An additional exploratory analysis was conducted to evaluate the effectiveness of new treatment with dual antiplatelet therapy following incident myocardial infarction. To overcome the influence of immortal time bias introduced by the use of community prescription records, exposure to dual antiplatelet therapy was included in the multivariable Cox regression model as a time-dependent covariate, with exposure occurring only after the first community prescription was dispensed. In brief, immortal time bias occurs due to the erroneous misclassification of exposure status when estimating the relationship between an exposure and an outcome.^23^ In the current study, we compared the hazard of experiencing cardiovascular death following incident myocardial infarction in those with kidney failure who received new treatment with dual antiplatelet therapy vs. those who did not (the primary exposure). Confounders were specified *a priori* and included age, sex, social deprivation status (SIMD quintile), comorbidities (prior heart failure, myocardial infarction, or stroke), primary kidney disease diagnosis, KRT modality at the time of the event, and concomitant treatment with ACE inhibitors/ARBs, beta-blockers, and statins post-discharge. All statistical analysis was performed in R, Version 3.5.1 (R Foundation for Statistical Computing, Vienna, Austria).

### Study approvals

The study was performed in agreement with the Declaration of Helsinki. The NHS Public Benefit and Privacy Panel for Health and Social Care and the Scottish Renal Registry steering committee independently approved the study and access to the data (Reference: 1819-0323). We did not seek individual patient consent as the study utilized fully anonymized data. Patient-level data can be requested following direct application to the NHS Public Health Scotland team. Our analysis code is publicly available here.

## Results

### Characteristics of patients with incident myocardial infarction and stroke

From 1996 to 2016, amongst 16 050 patients with kidney failure [52 (SD 15) years; 41.5% women], there were 1992 incident episodes of myocardial infarction and 996 incident episodes of stroke (*Table 1*). Patients with myocardial infarction were of a similar age to those with stroke [mean age 66 (SD 12) years vs. 65 (SD 13) years] but were more likely to be men (65.2% vs. 54.9%). Compared with patients with incident stroke, those with incident myocardial infarction were more likely to have a history of heart failure (20.2% vs. 13.2%), previous myocardial infarction (11.4% vs. 4.3%), and stroke (4.3% vs. 3.1%).

**Table 1:** Baseline characteristics of patients with kidney failure and incident myocardial infarction and stroke, in 3-year groups

	Myocardial infarction							Stroke						
	1996 to 1999	2000 to 2003	2004 to 2007	2008 to 2011	2012 to 2016^a^	Overall	*P*-value (test for trend)	1996 to 1999	2000 to 2003	2004 to 2007	2008 to 2011	2012 to 2016^a^	Overall	*P*-value (test for trend)
**No. of patients, *n***	**287**	**359**	**379**	**441**	**526**	**1992**		**155**	**191**	**198**	**191**	**261**	**996**	
**Non-fatal event**	147 (51.2)	220 (61.3)	251 (66.2)	332 (75.3)	407 (77.4)	**1357 (68.1)**	<.001	148 (95.5)	182 (95.3)	182 (91.9)	179 (93.7)	250 (95.8)	**941 (94.5)**	.394
**Age, years**	64 (11)	67 (11)	68 (12)	66 (12)	67 (12)	**66 (12)**	.020	63 (12)	63 (13)	65 (13)	68 (13)	66 (13)	**65 (13)**	<.001
**Sex**							.348							.497
Women	104 (36.2)	122 (34.0)	145 (38.3)	155 (35.1)	168 (31.9)	**694 (34.8)**		61 (39.4)	85 (44.5)	97 (49.0)	88 (46.1)	118 (45.2)	**449 (45.1)**	
Men	183 (63.8)	237 (66.0)	234 (61.7)	286 (64.9)	358 (68.2)	**1298 (65.2)**		94 (60.6)	106 (55.5)	101 (51.0)	103 (53.9)	143 (54.8)	**547 (54.9)**	
**SIMD quintile** ^b^							.566							.838
1 (most deprived)	85 (29.7)	93 (26.1)	107 (28.5)	111 (25.2)	149 (28.4)	**545 (27.5)**		42 (27.2)	48 (25.4)	56 (28.3)	47 (24.6)	63 (24.1)	**256 (25.8)**	
2	60 (21.0)	87 (24.4)	101 (26.9)	106 (24.0)	121 (23.1)	**475 (24.0)**		33 (21.4)	50 (26.5)	41 (20.7)	46 (24.1)	56 (21.5)	**226 (22.8)**	
3	62 (21.7)	71 (19.9)	63 (16.8)	95 (21.5)	121 (23.1)	**412 (20.8)**		28 (18.2)	38 (20.1)	33 (16.7)	38 (19.9)	51 (19.5)	**188 (18.9)**	
4	44 (15.4)	53 (14.9)	64 (17.1)	66 (15.0)	71 (13.5)	**298 (15.0)**		34 (22.1)	24 (12.7)	40 (20.2)	36 (18.8)	54 (20.7)	**188 (18.9)**	
5 (least deprived)	35 (12.2)	52 (14.6)	40 (10.7)	63 (14.3)	62 (11.8)	**252 (12.7)**		17 (11.0)	29 (15.3)	28 (14.1)	24 (12.6)	37 (14.2)	**135 (13.6)**	
**Previous medical conditions**														
Heart failure	80 (27.9)	85 (23.7)	81 (21.4)	69 (15.6)	87 (16.5)	**402 (20.2)**	<.001	17 (11.0)	29 (15.2)	31 (15.7)	22 (11.5)	32 (12.3)	**131 (13.2)**	.549
Myocardial infarction	43 (15.0)	46 (12.8)	54 (14.2)	32 (7.3)	52 (9.9)	**227 (11.4)**	.003	NA	NA	7 (3.5)	12 (6.3)	14 (5.4)	**43 (4.3)**	.278
Stroke	14 (4.9)	15 (4.2)	16 (4.2)	16 (3.6)	24 (4.6)	**85 (4.3)**	.935	NA	10 (5.2)	5 (2.5)	8 (4.2)	NA	**31 (3.1)**	.190
Previous coronary revascularization	9 (3.0)	30 (7.8)	19 (4.7)	34 (7.2)	46 (8.2)	**138 (6.5)**	.029	NA	NA	9 (4.5)	12 (6.3)	9 (3.4)	**39 (3.9)**	.275
**Renal history**														
**Primary kidney disease** ^b^							<.001							.055
Diabetic nephropathy	56 (19.6)	70 (19.5)	86 (22.8)	109 (24.7)	166 (31.6)	**487 (24.5)**		34 (21.9)	40 (20.9)	51 (25.8)	50 (26.2)	92 (35.2)	**267 (26.8)**	
Glomerulonephritis	53 (18.5)	56 (15.6)	46 (12.2)	81 (18.4)	76 (14.5)	**312 (15.7)**		29 (18.7)	36 (18.8)	29 (14.6)	23 (12.0)	36 (13.8)	**153 (15.4)**	
Interstitial nephritis	51 (17.8)	65 (18.1)	71 (18.8)	99 (22.4)	101 (19.2)	**387 (19.5)**		33 (21.3)	44 (23.0)	40 (20.2)	31 (16.2)	52 (19.9)	**200 (20.1)**	
Multisystem	76 (26.6)	104 (29.0)	93 (24.6)	89 (20.2)	110 (21.0)	**472 (23.7)**		33 (21.3)	37 (19.4)	44 (22.2)	53 (27.7)	42 (16.1)	**209 (21.0)**	
Unknown	50 (17.5)	64 (17.8)	82 (21.7)	63 (14.3)	72 (13.7)	**331 (16.6)**		26 (16.8)	34 (17.8)	34 (17.2)	33 (17.3)	39 (14.9)	**166 (16.7)**	
**First KRT modality** ^b^							.006							.011
Haemodialysis	204 (71.1)	281 (78.3)	311 (82.1)	336 (76.2)	408 (77.7)	**1540 (77.3)**		104 (67.1)	138 (72.3)	150 (75.8)	142 (74.3)	203 (77.8)	**737 (74.0)**	
Peritoneal dialysis	80 (27.9)	77 (21.4)	64 (16.9)	98 (22.2)	102 (19.4)	**421 (21.1)**		50 (32.3)	50 (26.2)	46 (23.2)	45 (23.6)	50 (19.2)	**241 (24.2)**	
Kidney transplant	NA	NA	NA	NA	NA	**11 (0.6)**		NA	NA	NA	NA	NA	**11 (1.1)**	
**KRT modality at event**							<.001							<.001
Haemodialysis	156 (54.4)	255 (71.0)	296 (78.1)	316 (71.7)	395 (75.1)	**1418 (71.2)**		89 (57.4)	105 (55.0)	143 (72.2)	139 (72.8)	174 (66.7)	**650 (65.3)**	
Peritoneal dialysis	73 (25.4)	56 (15.6)	39 (10.3)	33 (7.5)	31 (5.9)	**232 (11.6)**		36 (23.2)	41 (21.5)	24 (12.1)	18 (9.4)	26 (10.0)	**145 (14.6)**	
Kidney transplant	58 (20.2)	48 (13.4)	44 (11.6)	92 (20.9)	100 (19.0)	**342 (17.2)**		30 (19.4)	45 (23.6)	31 (15.7)	34 (17.8)	61 (23.4)	**201 (20.2)**	

Values are *n* (%) or mean (SD).

SIMD, Scottish index of multiple deprivation.

^a^Patients included in this column are grouped from four calendar years. NA represents redacted data that might be considered potentially identifiable, including count data ≤5.

^b^Social deprivation status (SIMD) was missing in 0.4% and 0.3%, primary kidney disease was missing in 0.1% and 0.1%, and KRT modality was missing in 1.0% and 0.7% of all patients with myocardial infarction and stroke, respectively.

For patients with both incident myocardial infarction and stroke, the most common underlying cause of kidney disease was diabetic nephropathy [24.4% (487/1992) and 26.8% (267/996), respectively]. Whereas the proportion of patients with diabetic nephropathy increased between 1996 and 2016, other aetiologies of kidney disease remained relatively constant or decreased (see Supplementary data online, *Figure S2*). At the time of the incident event, haemodialysis was the most common KRT modality, whilst the proportion of patients receiving peritoneal dialysis decreased over time for both groups (*Table 1*; Supplementary data online, *Figure S3*). Patients receiving maintenance haemodialysis at the time of incident myocardial infarction and stroke were the oldest and most socially deprived, whilst those with a kidney transplant were the youngest (see Supplementary data online, *Table S2*).

### Trends in the incidence of myocardial infarction and stroke

**Figure 1:**
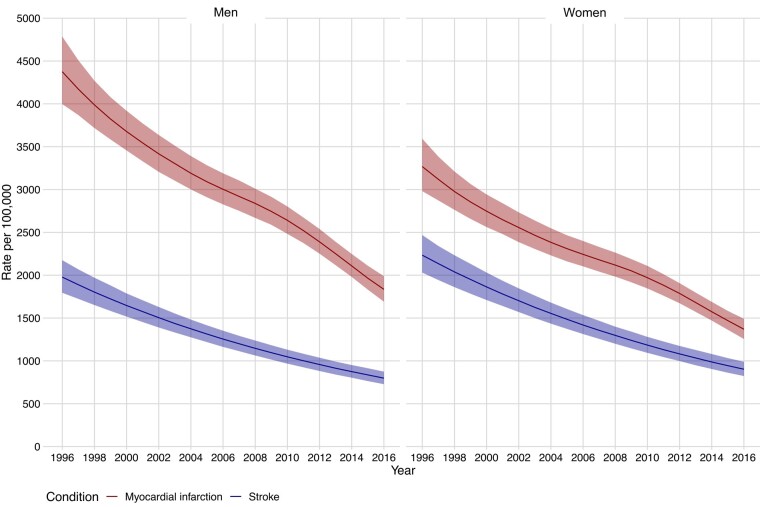
Age-standardized incidence of myocardial infarction and stroke in patients with kidney failure in Scotland between 1996 and 2016, stratified by sex

In patients with kidney failure, the age-standardized incidence of myocardial infarction and stroke decreased in both sexes between 1996 and 2016. For myocardial infarction, this decreased from 4376 [95% confidence interval (CI) 3998–4785] to 1835 (95% CI 1692–1988) per 100 000 in men, and from 3268 (95% CI 2982–3593) to 1369 (95% CI 1257–1491) in women (*Figure 1*; Supplementary data online, *Table S3*). For stroke, age-standardized incidence decreased from 1978 (95% CI 1795–2175) to 799 (95% CI 729–875) per 100 000 in men, and from 2234 (95% CI 2031–2468) to 903 (95% CI 824–990) in women (*Figure 1*; Supplementary data online, *Table S3*).

Compared with younger patients, older patients with kidney failure experienced a greater fall in these incidence rates (see Supplementary data online, *Figure S4*). When stratified by KRT modality at the time of the incident event, patients with a kidney transplant had the lowest incidence of myocardial infarction and stroke (see Supplementary data online, *Figure S5*). Between 1996 and 2016, the incidence of myocardial infarction and stroke in those with a kidney transplant was stable, in contrast to those on maintenance haemodialysis, whose incidence rates were declining.

### Trends in the incidence of myocardial infarction and stroke in patients with kidney failure compared with the general population

**Figure 2:**
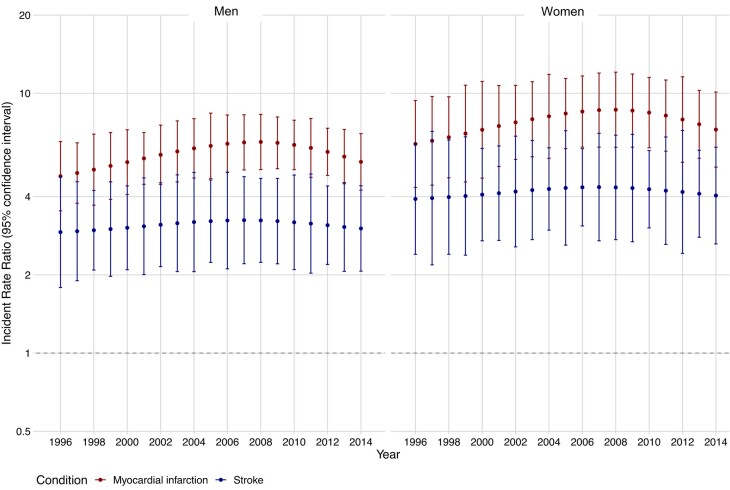
Incidence rate ratios for myocardial infarction and stroke between 1996 and 2014, stratified by sex

Compared with the general population, patients with kidney failure had a significantly higher incidence of myocardial infarction and, to a lesser extent, stroke (*Figure 2*). When stratified by sex, the IRRs for myocardial infarction and stroke showed a trend towards being higher in women with kidney failure, but with considerable overlap in the 95% CI. Over time, comparing women with kidney failure with women in the general population, the IRR for myocardial infarction was 6.38 (95% CI 4.34–9.38) in 1996 and 7.25 (95% CI 5.20–10.11) in 2014. In men, the IRR was 4.80 (95% CI 3.53–6.52) in 1996 and 5.45 (95% CI 4.24–6.99) in 2014. For stroke, the changes in IRR were more modest. For example, in women, the IRR for stroke was 3.92 (95% CI 2.40–6.40) in 1996 and 4.04 (95% CI 2.63–6.20) in 2014, whilst in men it was 2.92 (95% CI 1.79–4.77) in 1996 and 3.02 (95% CI 2.07–4.41) in 2014.

When stratified by age, the incidence of myocardial infarction was elevated to a similar extent in younger and older patients with kidney failure compared with the general population (see Supplementary data online, *Figure S6*). In contrast, younger patients with kidney failure had an especially high risk of stroke when compared with the general population. When stratified by KRT modality, patients on haemodialysis had the highest incidence of myocardial infarction, whilst those with a kidney transplant had only a modestly increased incidence of myocardial infarction relative to the general population (see Supplementary data online, *Figure S7*). Comparable trends were evident for stroke according to KRT modality, although patients with a kidney transplant had a similar incidence of stroke to that of the general population.

### Trends in cardiovascular therapies following incident myocardial infarction and stroke

Individual patient-level prescribing data were available from 2009 until 2016 (see Supplementary data online, *Table S4* and Supplementary data online, *Figure S8*). Common preventative therapies were prescribed in around half of patients with kidney failure at the time of their incident event. For example, aspirin was prescribed in 58.3% and 53.8% of all patients at the time of myocardial infarction and stroke, respectively. The proportion of patients prescribed preventative therapies was stable between 2009 and 2016, except for beta-blockers, the prescription of which increased from 52.5% to 64.2% for patients who went on to have a myocardial infarction, and from 42.3% to 58.2% for patients who went on to have a stroke. Following non-fatal incident myocardial infarction, the overall proportion of patients prescribed aspirin or clopidogrel increased. In addition, the proportion of patients prescribed dual antiplatelet therapy increased over time, from 40.7% to 61.4%, with most patients (94.1%; 337/358) prescribed aspirin and clopidogrel. Following incident non-fatal stroke, the proportion of patients prescribed aspirin decreased during this period from 49.0% to 26.4%, whilst the proportion prescribed clopidogrel increased from 14.6% to 53.8% (see Supplementary data online, *Table S4*; Supplementary data online, *Figure S8*).

### Patient outcomes following incident myocardial infarction and stroke

Crude outcomes following incident myocardial infarction and stroke in patients with kidney failure between 1996 and 2016 are summarized in *Table 2*. During this period, both short- and longer term outcomes for these patients were poor. The primary outcome of cardiovascular death at 1 year occurred in 61.1% (1217/1992) and 52.5% (523/996) of patients with myocardial infarction and stroke, respectively. There was a decline in mortality over this 20-year period for both conditions. However, for those with myocardial infarction, this was accompanied by an increase in subsequent non-fatal cardiovascular events, including heart failure, bleeding hospitalizations, and recurrent myocardial infarction (*Table 2*). Compared with myocardial infarction, those with stroke remained hospital inpatients for longer.

**Table 2:** Crude outcomes at 1 and 3 years for patients with kidney failure and incident myocardial infarction and stroke, in 3-year groups

	Myocardial infarction							Stroke						
	1996 to 1999	2000 to 2003	2004 to 2007	2008 to 2011	2012 to 2016^a^	Overall	*P*-value (test for trend)	1996 to 1999	2000 to 2003	2004 to 2007	2008 to 2011	2012 to 2016^a^	Overall	*P*-value (test for trend)
**No. of patients, *n***	**287**	**359**	**379**	**441**	**526**	**1992**		**155**	**191**	**198**	**191**	**261**	**996**	
**Length of stay, days**	3.00 [1.00, 7.00]	3.00 [1.00, 9.50]	5.00 [1.50, 10.00]	3.00 [1.00, 7.00]	3.00 [1.00, 7.00]	**3.00 [1.00, 8.00]**	<.001	7.00 [2.00, 18.50]	7.00 [3.00, 20.50]	6.00 [2.00, 15.00]	8.00 [3.00, 16.50]	5.00 [2.00, 14.00]	**6.00 [2.00, 17.00]**	.095
**Cardiovascular death**														
30 days	193 (67.2)	222 (61.8)	196 (51.7)	177 (40.1)	188 (35.7)	**976 (49.0)**	<.001	62 (40.0)	60 (31.4)	73 (36.9)	64 (33.5)	68 (26.1)	**327 (32.8)**	.030
1 year	215 (74.9)	266 (74.1)	248 (65.4)	230 (52.2)	258 (49.0)	**1217 (61.1)**	<.001	98 (63.2)	105 (55.0)	102 (51.5)	105 (55.0)	113 (43.3)	**523 (52.5)**	.002
3 years	228 (79.4)	300 (83.6)	282 (74.4)	280 (63.5)	345 (65.6)	**1435 (72.0)**	<.001	114 (73.5)	120 (62.8)	126 (63.6)	131 (68.6)	154 (59.0)	**645 (64.8)**	.030
**All-cause death**														
30 days	193 (67.2)	222 (61.8)	197 (52.0)	177 (40.1)	190 (36.1)	**979 (49.1)**	<.001	63 (40.6)	62 (32.5)	75 (37.9)	67 (35.1)	70 (26.8)	**337 (33.8)**	.030
1 year	219 (76.3)	271 (75.5)	258 (68.1)	244 (55.3)	274 (52.1)	**1266 (63.6)**	<.001	105 (67.7)	112 (58.6)	112 (56.6)	116 (60.7)	127 (48.7)	**572 (57.4)**	.003
3 years	234 (81.5)	309 (86.1)	301 (79.4)	307 (69.6)	380 (72.2)	**1531 (76.9)**	<.001	127 (81.9)	137 (71.7)	144 (72.7)	146 (76.4)	177 (67.8)	**731 (73.4)**	.025
**Non-fatal events** ^a^														
**Bleeding**														
1 year	NA	NA	14 (5.6)	21 (6.3)	23 (5.7)	**68 (5.0)**	.028	11 (7.4)	20 (11.0)	13 (7.1)	13 (7.3)	12 (4.8)	**69 (7.3)**	.202
3 years	10 (6.8)	10 (4.5)	28 (11.2)	36 (10.8)	52 (12.8)	**136 (10.0)**	<.001	15 (10.1)	26 (14.3)	27 (14.8)	18 (10.1)	27 (10.8)	**113 (12.0)**	.493
**Heart failure**														
1 year	6 (4.1)	16 (7.3)	26 (10.4)	48 (14.5)	64 (15.7)	**160 (11.8)**	<.001	7 (4.7)	9 (4.9)	NA	NA	12 (4.8)	**38 (4.0)**	.569
3 years	12 (8.2)	23 (10.5)	37 (14.7)	62 (18.7)	81 (19.9)	**215 (15.8)**	<.001	11 (7.4)	13 (7.1)	10 (5.5)	9 (5.0)	21 (8.4)	**64 (6.8)**	.582
**Myocardial infarction**														
1 year	29 (19.7)	35 (15.9)	45 (17.9)	108 (32.5)	160 (39.3)	**377 (27.8)**	<.001	NA	7 (3.8)	10 (5.5)	NA	14 (5.6)	**36 (3.8)**	.086
3 years	30 (20.4)	43 (19.5)	59 (23.5)	122 (36.7)	182 (44.7)	**436 (32.1)**	<.001	NA	NA	16 (8.8)	8 (4.5)	17 (6.8)	**52 (5.5)**	.050
**Stroke**														
1 year	NA	NA	12 (4.8)	NA	9 (2.2)	**30 (2.2)**	.028	31 (20.9)	36 (19.8)	26 (14.3)	35 (19.6)	32 (12.8)	**160 (17.0)**	.105
3 years	6 (4.1)	7 (3.2)	18 (7.2)	7 (2.1)	17 (4.2)	**55 (4.1)**	.047	33 (22.3)	45 (24.7)	36 (19.8)	39 (21.8)	41 (16.4)	**194 (20.6)**	.283
**Subsequent coronary revascularization**														
1 year	NA	NA	NA	NA	NA	**9 (0.7)**	.559							
3 years	NA	NA	NA	7 (2.1)	13 (3.2)	**31 (2.3)**	.293							

Values are median IQR] or *n* (%).

^a^The denominator for these percentages is derived from the number of non-fatal events in that year category. NA represents redacted data that might be considered potentially identifiable, including count data ≤5.

Crude outcomes were then compared amongst patients with different KRT modalities at the time of the incident event. For both myocardial infarction and stroke, cardiovascular death at 1 year in those receiving maintenance dialysis (haemodialysis or peritoneal dialysis) was almost two-fold higher than in patients with a kidney transplant (see Supplementary data online, *Table S5*). In contrast, patients with a kidney transplant had the highest rates of recurrent myocardial infarction and recurrent stroke.

**Figure 3:**
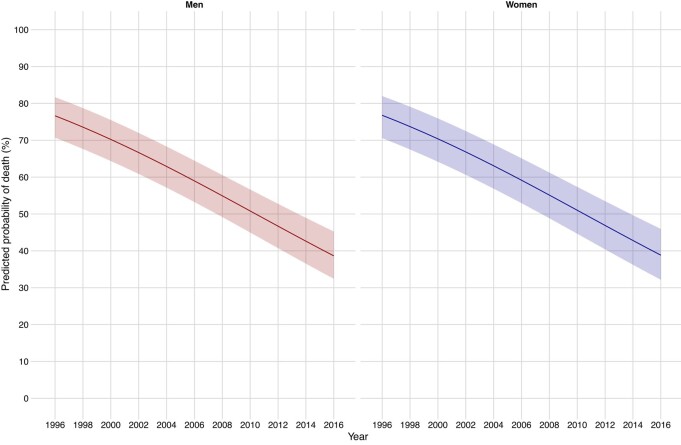
Predicted probability of cardiovascular case fatality at 1 year incident myocardial infarction in men and women with kidney failure between 1996 and 2016

Trends in cardiovascular death at 1 year following incident myocardial infarction and stroke, stratified by sex, and adjusted for age, social deprivation, and comorbidities, are presented in *Figure 3* and Supplementary data online, *Table S6*. For a 66-year-old man with kidney failure (the mean age of our cohort), the predicted probability of cardiovascular death within 1 year of a myocardial infarction decreased from 76.6% (95% CI 70.7%–81.7%) in 1996 to 38.6% (95% CI 32.4%–45.2%) in 2016, whilst for a 66-year-old woman it decreased from 76.8% (95% CI 70.6%–82.0%) to 38.8% (95% CI 32.2%–45.9%) (*Figure 3*; Supplementary data online, *Table S6*). Reductions in cardiovascular and all-cause death at 1 year were similar irrespective of KRT modality at the time of the incident event and were lowest in patients with a kidney transplant (see Supplementary data online, *Figures S9* and *S10*).

Cardiovascular death following incident stroke also improved over time for both men and women with kidney failure, but to a lesser extent than for myocardial infarction. The predicted probability of cardiovascular death within 1 year for a 66-year-old man with kidney failure decreased from 63.5% (95% CI 53.9%–72.1%) in 1996 to 41.4% (95% CI 32.5%–50.9%) in 2016, and from 67.6% (95% CI 58.0%–75.9%) to 45.8% (95% CI 36.7%–55.3%) for a 66-year-old woman (*Figure 3*; Supplementary data online, *Table S6*). Again, similar reductions in cardiovascular and all-cause death at 1 year were observed across all KRT modalities, with the lowest rates in patients with a kidney transplant (see Supplementary data online, *Figures S9* and *S10*).

### Effectiveness of dual antiplatelet therapy following incident myocardial infarction in patients with kidney failure

Following incident myocardial infarction, patients who were newly prescribed dual antiplatelet therapy were less likely to die of cardiovascular causes within 1 year than those who were not [13.6% (41/301) vs. 40.5% (75/185)]. After adjustment for confounding variables and including exposure to new dual antiplatelet therapy as a time-dependent covariate, this treatment effect at 1 year persisted [adjusted hazard ratio (aHR) 0.69, 95% CI 0.53–0.90, *P* = .006]. The proportion of patients experiencing a subsequent bleeding event within 1 year of incident myocardial infarction was slightly higher in those who were newly prescribed dual antiplatelet therapy [7.6% (23/301) vs. 4.9% (9/185)], although this difference may be partly explained by the competing risk of cardiovascular death in those who did not receive new dual antiplatelet therapy.

## Discussion

We performed a national population-based data linkage study to evaluate trends in the incidence, treatment, and outcomes of myocardial infarction and stroke in patients with kidney failure over the last 20 years. We make several important observations that could inform future healthcare provision for this high-risk population. First, although the incidence rates of both myocardial infarction and stroke have halved in patients with kidney failure, these remain substantially higher compared with the general population. When stratified by KRT modality, the biggest reductions in the incidence of both events were observed in patients on haemodialysis, whilst incidence rates in those with a kidney transplant were lower and stable. Second, there was an ∼1.5-fold increase in the prescription of dual antiplatelet therapy following a myocardial infarction and an ∼3-fold increase in the prescription of antiplatelet therapy following a stroke over the study period. Third, although cardiovascular death following myocardial infarction and stroke has reduced substantially over the past 20 years in both men and women with kidney failure and across all KRT modalities, overall outcomes remain poor (*Structured Graphical Abstract*).

Our study has several strengths. We utilized established, high-quality linked healthcare datasets to define the study population and outcomes, and to facilitate near-complete patient follow-up. These data assets have been used to deliver numerous high-quality observational^9,18,21,24–26^ and randomized^27–29^ trials, and have demonstrated high accuracy and completeness.^18,30^ Our study design minimized selection bias by ensuring all patients with kidney failure were identified and included through linkage with the Scottish Renal Registry, and by identifying all consecutive myocardial infarction and stroke events during study follow-up through patient-level linkage with a national hospitalization dataset. Finally, patients with kidney failure living in Scotland are comparable with those living in other European countries and in the USA,^16,31,32^ making our results broadly generalizable.

To date, no study has evaluated temporal trends in the incidence of fatal and non-fatal myocardial infarction and stroke in patients with kidney failure, reported these rates according to age, sex, and KRT modality, and compared these with event rates in the general population. Although the incidence of myocardial infarction has halved over the last 20 years in this at-risk group, the incidence was still about four- to eight-fold higher than in the general population. These findings are consistent with those of O’Lone and colleagues, who reported an incidence of myocardial infarction of 2072 per 100 000 person-years between 2000 and 2010 in patients with kidney failure in Australia and New Zealand.^12^ Using the United States Renal Data System (USRDS), others have shown that the incidence of myocardial infarction in patients receiving haemodialysis reached a peak of 8080 per 100 000 person-years in 2002, before falling to 7310 per 100 000 person-years in 2011.^33^ Whilst these absolute rates are two-fold higher than we observed in patients receiving haemodialysis in the present study, the temporal trend is similar. Although two studies (published >20 years ago) evaluated the incidence of myocardial infarction in kidney failure, they are significantly limited by restricting their findings to patients on haemodialysis and not reporting temporal trends.^34,35^

When stratified by KRT modality, patients with a kidney transplant included in our study had the lowest incidence of myocardial infarction and stroke compared with other KRT modalities. In addition, the incidence of these conditions was stable in patients with kidney transplant rather than declining—as was the case for patients on haemodialysis or in the general population. The lower incidence of myocardial infarction and stroke in patients with a kidney transplant can partly be explained by the fact that kidney transplantation is the single most effective intervention to reduce cardiovascular risk in patients with kidney failure.^36^ Moreover, patients who undergo kidney transplantation are generally younger, less multimorbid, less frail, and more fit than those who remain on peritoneal dialysis or haemodialysis.^37^ Therefore, due to their lower overall cardiovascular risk, it is perhaps unsurprising that the incidence of myocardial infarction and stroke is lower in patients with a kidney transplant. One potential explanation of why the incidence of myocardial infarction and stroke has failed to decline in recent years in those with a kidney transplant is the change in the demographic of patients undergoing kidney transplantation and changes in clinical practice over time. For example, in recent years, there has been a relative increase in kidney transplant recipient age, body mass index, proportion with underlying diabetes and a history of prior kidney transplant, and length of time on peritoneal dialysis or haemodialysis,^38^ all of which increase the baseline cardiovascular risk of patients with a kidney transplant.

Similar to myocardial infarction, the few studies that have examined stroke incidence in kidney failure have been restricted to patients on dialysis, have lacked prescribing data, or have not reported temporal trends according to age, sex, or KRT modality.^11^ Nevertheless, the incidence of stroke in patients with kidney failure we describe here is in keeping with this earlier work.^13,39^ Additionally, the reduction in stroke incidence we observed over our 20-year study period is in line with the trend reported by Alqahtani *et al*., who described a fall from 1390 to 783 per 100 000 between 2003 and 2014 in patients receiving dialysis.^40^ Despite this, Alqahtani and colleagues estimated the overall incidence of stroke in patients on maintenance dialysis to be about eight-fold higher than in the general population, which is substantially greater than the ratio estimated here. This may be partly related to the observation noted by Alqahtani and colleagues that approximately one-third of patients on dialysis in their study cohort were black.^40^ In contrast, only ∼4% of the overall Scottish population is non-white.^41^

In the general population, incidence rates of myocardial infarction and stroke have declined approximately four-fold over the past three decades.^9^ We observed far smaller reductions for patients with kidney failure. This may be because the attributable risk of traditional risk factors—and the prescription and overall effectiveness of pharmacotherapies for primary prevention—is lower in patients with kidney failure relative to the general population.^42^ Cardiovascular risk prediction in patients with kidney failure is complicated. It relates in part to traditional cardiovascular risk factors, but also to the adverse effects of systemic inflammation, disordered calcium metabolism, chronic anaemia, and structural alterations in the myocardium.^43^ In the present study, cardiovascular preventative therapies were prescribed in around half of all patients at the time of their incident event. Following myocardial infarction and stroke, we generally observed little change in the proportion of patients with kidney failure prescribed beta-blockers, statins, and ACE inhibitors, perhaps partly explaining why outcomes remain poorer compared with the general population. However, there are limited data on the effectiveness of such preventative therapies in patients with kidney disease—especially in those with kidney failure—due to the exclusion of these patients from cardiovascular trials.^44^

Our study is the first to report prescribing data before and after a myocardial infarction or stroke in patients with kidney failure. Interestingly, by the end of our study, we found that two in three patients with kidney failure were prescribed dual antiplatelet therapy following a myocardial infarction and antiplatelet therapy following a stroke. These represent ∼1.5-fold and ∼3-fold increases over a 7-year period, respectively. Overall, these trends are consistent with—though less impressive than—the increased uptake of pharmacotherapies and interventions following cardiovascular events in patients with less severe kidney disease or normal kidney function.^28,45^ Nevertheless, these prescribing trends are encouraging and may partly explain some of the improvements we observed in cardiovascular death at 1 year following myocardial infarction and stroke. However, we were unable to determine to what extent other factors, such as improvements in dialysis and transplant care or in the management of non-traditional risk factors such as chronic anaemia,^46^ have also contributed to improved outcomes of patients with kidney failure.

We performed an exploratory analysis to investigate the effectiveness of dual antiplatelet therapy following incident myocardial infarction in patients with kidney failure. After adjustment for confounding variables and including exposure to dual antiplatelet therapy as a time-dependent covariate to minimize the influence of immortal time bias, patients with kidney failure who were prescribed dual antiplatelet therapy following incident myocardial infarction had a 30% lower risk of cardiovascular death at 1 year compared with those who were not prescribed dual antiplatelet therapy following their incident event. Whilst these are promising results, there remains an urgent need for randomized trials to robustly evaluate both the safety and effectiveness of these therapies in patients with kidney failure.

Over the 20-year study period, outcomes of patients with kidney failure improved significantly following myocardial infarction and stroke, even after adjustment for age, social deprivation status, and comorbidities. This is in line with the findings of Ocak and colleagues,^16^ who noted reductions in death following myocardial infarction and stroke that were greater in patients receiving maintenance dialysis than in the general population. Using USRDS data, Shroff *et al.* described crude outcomes that were marginally better than those reported here.^14^ The authors attributed these findings to a marked increase in percutaneous coronary intervention rates. This contrasts with our study, where coronary revascularization rates following the incident event were exceptionally low (<1% at 1 year; 2.3% at 3 years). Nevertheless, as Szummer and colleagues previously showed, the apparent benefit of an early invasive strategy following myocardial infarction in patients with kidney failure is unclear,^47^ and has yet to be addressed in a randomized trial. Of note, for patients with incident myocardial infarction, we also observed an increase in subsequent non-fatal cardiovascular complications, including heart failure and recurrent myocardial infarction, during the study period. Whilst these trends are likely to be a consequence of improved patient survival, they have important implications for future healthcare provision in this at-risk population.

We recognize some limitations to our work. Whilst the Scottish Renal Registry has 100% unit and patient coverage,^48^ we relied on routinely collected, administrative ICD-9/-10 codes from hospitalizations and administrative death records to identify incident episodes of myocardial infarction and stroke. To minimize the impact of case ascertainment bias, we utilized a previously validated approach and only included episodes of myocardial infarction and stroke based on the identification of relevant administrative codes in the first two positions of hospitalization and death records.^18^ Our study population was also restricted to patients with kidney failure, and excluded other important patient groups such as those with stage IV chronic kidney disease (estimated glomerular filtration rate 15–29 mL/min/1.73 m^2^). These patients are at a high risk of cardiovascular and renal complications, and will be an important group to include in future work in this area. The national prescribing dataset was only available from 2009 onwards and was restricted to medications dispensed in the community. Thus, we were unable to evaluate earlier prescribing trends, neither were we able to describe trends in the prescription of in-hospital treatments, such as thrombolysis for acute ischaemic stroke. However, ours is the first study to report prescribing data before and after a myocardial infarction or stroke in patients with kidney failure. We were also unable to include data relating to smoking status and body mass index due to a high rate of missingness affecting these variables, and thus our analyses are subject to unmeasured confounding. In addition, we acknowledge the potential impact of confounding-by-indication on the results of our exploratory analysis evaluating the effectiveness of dual antiplatelet therapy following incident myocardial infarction in patients with kidney failure. Finally, the mean age of our study cohort is younger than reported in other national registries from a similar time period.^49^ This is, in part, because patients with kidney failure not receiving KRT (termed ‘conservative care’) were not included in the present study. These patients tend to be older and more multimorbid than those with kidney failure currently receiving KRT.^50^ Data relating to these patients are now being collected by the Scottish Renal Registry for the purposes of research, meaning outcomes for this patient group will be reported in similar studies in the future.

## Conclusions and future directions

There has been a significant decline in the incidence of myocardial infarction and stroke in patients with kidney failure over the past 20 years, yet these rates remain substantially elevated compared with the general population. The reasons for this are unclear, and therefore future work should focus on exploring the changing burden of both traditional and non-traditional cardiovascular risk factors in these high-risk patients. Despite improvements in patient outcomes during the study period, the prognosis of patients with kidney failure who experience a myocardial infarction or stroke remains poor. In part, these poor outcomes may be driven by the therapeutic disenfranchisement of patients with kidney failure, which is likely to be a consequence of the systematic exclusion of patients with kidney failure from cardiovascular trials. In summary, our contemporaneous findings provide a crucial framework to inform public health policy for patients with kidney failure, and set the scene for clinical trials that robustly evaluate the efficacy and safety of cardiovascular therapies specifically in this high-risk patient population.

## Supplementary Material

ehae080_Supplementary_Data

## Declarations

### Data Availability

Patient-level data can be requested following direct application to the NHS Public Health Scotland team.
