# Supplementary material for: Kidney replacement therapy: trends in incidence, treatment, and outcomes of myocardial infarction and stroke in a nationwide Scottish study
Source: Eur Heart J. 2024 Mar 1;45(15):1339–51. doi: 10.1093/eurheartj/ehae080 (PMC11015953; doi:10.1093/eurheartj/ehae080)
Supplement: ehae080_Supplementary_Data [file ehae080_supplementary_data.pdf]

SUPPLEMENTARY MATERIAL

Kidney replacement therapy: trends in incidence, treatment, and outcomes of myocardial infarction and stroke in a nationwide Scottish study

Peter J. Gallacher, MBChB MPH,[1] David Yeung, BSc,[1] Samira Bell, MBChB MD,[2,3] Anoop SV Shah, MBChB PhD,[4] Nicholas L Mills, MBChB PhD,[1,5] Neeraj Dhaun, MBChB PhD[1,6]

[1] BHF/University Centre for Cardiovascular Science, University of Edinburgh, Edinburgh, UK

[2] Division of Population Health and Genomics, University of Dundee, Dundee, UK

[3] Scottish Renal Registry, Scottish Health Audits, Public Health Scotland, Glasgow, UK

[4] Department of Non-Communicable Epidemiology, London School of Hygiene and Tropical Medicine, London, UK

[5] Usher Institute, University of Edinburgh, Edinburgh, UK

[6] Department of Renal Medicine, Royal Infirmary of Edinburgh, Edinburgh, UK

Corresponding author:

Dr. Neeraj Dhaun *(Bean)*

BHF/University Centre for Cardiovascular Science

University of Edinburgh

Edinburgh EH16 4SA

United Kingdom

E-mail: bean.dhaun@ed.ac.uk

Supplementary text 1

Data sources

Scottish Renal Registry

The Scottish Renal Registry is a national registry of all patients receiving kidney replacement therapy (KRT) (i.e., HD, peritoneal dialysis, and transplant) for kidney failure for ≥90 days in Scotland. It collates data from all nine adult renal units in Scotland and 28 satellite HD units, serving a population of 5.4 million people. The Scottish Renal Registry was established in 1991 and backfilled to 1960 with data from the European Renal Association–European Dialysis and Transplant Association (ERA-EDTA; the first patient was dialyzed for kidney failure in Scotland in 1960). It has 100% unit and patient coverage.

Scottish Morbidity Record 01

This patient-level dataset collates all acute, inpatient, and day-case hospitalizations in Scotland from 1981 onwards. The primary condition is coded according to WHO International Classification of Diseases (ICD) 9/10 codes. Surgical procedures are coded according to the Office of Population, Censuses and Surveys (OPCS) classification. SMR01 data are considered amongst the best routinely collected healthcare data worldwide, in terms of granularity, population coverage and linkage capabilities. Across all SMR01 records, estimated completion and accuracy rates are 99% and 89%, respectively.[1] For SMR01 records relating to cardiovascular disease, the accuracy rate is 94.2%.[2]

National Records of Scotland

This patient-level dataset includes all deaths in Scotland registered annually from 1982 onwards.[3] Linkable data include patient demographics, and primary and secondary causes of death.

Prescribing Information Service

Prescriptions Information Service holds data regarding every prescription dispensed in the community from 2009.[4] The dataset includes information about the dispensed drug, the patient, the prescriber, and the dispenser. *Prescriptions Information Service* does not hold information regarding prescriptions dispensed from in-hospital pharmacies or regarding prescriptions that are not collected by patients in the community. The completeness and accuracy of this dataset are expected to be high because dispensers do not receive payment for prescriptions until this information is submitted.

Supplementary table 1. List of ICD-9/-10 codes employed in study. *Abbreviations: BNF: British National Formulary; ICD: international classification of diseases; OPCS-4: Office of Population, Censuses and Surveys 4. *For chronic respiratory disease, the presence of a single ICD-10 code in SMR01 data **OR** a single BNF code in community prescription data in the 365 days preceding the incident event was sufficient to identify this comorbidity.*

Condition	ICD-9/-10 or BNF code
**Chronic respiratory disease***	
ICD-10	I27.8, I27.9 J40-45, J46, J47, J60-67, J68.4, J70.1, J70.3
BNF	030101, 030102, 030104, 0302, 030302
**Diabetes mellitus**	
BNF	060101, 060102
**Gastro-esophageal reflux disease**	
BNF	010101, 010102, 010301, 010305
**Gout**	
BNF	100104
**Cardiovascular conditions**	**ICD-9/-10 code**
**Bleeding event/procedure**	
ICD-9	5307, 5693, 5780, 5781, 5789, 6207, 6238, 6239, 6341, 6341, 6351, 6351, 6361, 6361, 6371, 6371, 6381, 6381, 6391, 6408, 6409, 641, 6413, 6418, 6419, 6657, 6645, 6743, 7863, 7863, 7863, 9981, 5310, 5314, 5320, 5324, 5330, 5334, 5340, 5344, 5350, 5354, 641, 6413, 6418, 6419, 6660, 6661, 6662, 7703, 7703, 7703, 7703, 7848, 7863, 7863, 7863, 4560, 5312, 5316, 5322, 5326, 5332, 5336, 5342, 5346, 3628, 3792, 430, 432, 438, 438, 8520, 8520, 8520, 4230
ICD-10	K226, K625, K920, K921, K922, N837, N938, N939, O031, O036, O041, O046, O051, O056, O061, O066, O071, O076, O081, O208, O209, O46, O460, O468, O469, O717, O717, O902, R042, R048, R049, T810, K250, K254, K260, K264, K270, K274, K280, K284, K290, K291, O67, O670, O678, O679, O720, O721, O722, P260, P261, P268, P269, R041, R042, R048, R049, I850, K252, K256, K262, K266, K272, K276, K282, K286, H356, H431, H450, I60, I62, I690, I692, S064, S065, S066, I230, I312
OPCS-4	E05, E058, E059, F162, D041, E203, F365, G523, H212, H531, K681, P093, P271, T301, Y221, T032, Y321, V032, A052, A053, A054, A103, A401, A411
**Coronary revascularization**	
OPCS-4	K25 - K35, K40 – 46, K49, K50, K63, K65, K75, U102, U105
**Heart failure**	
ICD-9	428
ICD-10	I50
**Myocardial infarction**	
ICD-9	413

ICD-10	I21, I22
**Stroke**	
ICD-9	430 - 438
ICD-10	G45, G46, I60-69

Supplementary table 2. Baseline characteristics of patients with kidney failure and incident myocardial infarction (left panel) and stroke (right panel) between 1996 and 2016, grouped by KRT modality at time of event.

	Myocardial infarction					Stroke				
	Hemodialysis	Peritoneal dialysis	Kidney transplant	Overall	P-value (test for trend)	Hemodialysis	Peritoneal dialysis	Kidney transplant	Overall	P-value (test for trend)
**Number of patients, n**	**1,418**	**232**	**342**	**1,992**	**-**	**650**	**145**	**201**	**996**	**-**
**Non-fatal event**	935 (65.9)	146 (62.9)	276 (80.7)	**1,357 (68.1)**	<0.001	604 (92.9)	140 (96.6)	197 (98.0)	**941 (94.5)**	0.011
**Age, years**	68 (11)	66 (10)	58 (11)	**66 (12)**	<0.001	67 (13)	64 (14)	59 (12)	**65 (13)**	<0.001
**Sex**					0.037					0.021
Women	518 (36.5)	75 (32.3)	101 (29.5)	**694 (34.8)**	-	299 (46.0)	75 (51.7)	75 (37.3)	**449 (45.1)**	-
Men	900 (63.5)	157 (67.7)	241 (70.5)	**1,298 (65.2)**	-	351 (54.0)	70 (48.3)	126 (62.7)	**547 (54.9)**	-
**SIMD quintile§**					0.017					0.509
1 (most deprived)	419 (29.7)	47 (20.3)	79 (23.2)	**545 (27.5)**	-	182 (28.1)	34 (23.6)	40 (19.9)	**256 (25.8)**	-
2	331 (23.5)	52 (22.5)	92 (27.0)	**475 (24.0)**	-	140 (21.6)	35 (24.3)	51 (25.4)	**226 (22.8)**	-
3	279 (19.8)	63 (27.3)	70 (20.5)	**412 (20.8)**	-	118 (18.2)	31 (21.5)	39 (19.4)	**188 (18.9)**	-
4	200 (14.2)	41 (17.7)	57 (16.7)	**298 (15.0)**	-	123 (19.0)	26 (18.1)	39 (19.4)	**188 (18.9)**	-
5 (least deprived)	181 (12.8)	28 (12.1)	43 (12.6)	**252 (12.7)**	-	85 (13.1)	18 (12.5)	32 (15.9)	**135 (13.6)**	-
**Previous medical conditions**										
Heart failure	345 (24.3)	44 (19.0)	13 (3.8)	**402 (20.2)**	<0.001	110 (16.9)	NA	NA	**131 (13.2)**	<0.001

Myocardial infarction	182 (12.8)	32 (13.8)	13 (3.8)	**227 (11.4)**	<0.001	34 (5.2)	NA	NA	**43 (4.3)**	0.074
Stroke	70 (4.9)	7 (3.0)	8 (2.3)	**85 (4.3)**	0.062	25 (3.8)	NA	NA	**31 (3.1)**	0.180
Previous coronary revascularization	97 (6.8)	18 (7.8)	7 (2.0)	**138 (6.5)**	0.002	28 (4.3)	NA	NA	**39 (3.9)**	0.277
**Renal history**										
**Primary kidney disease§**					<0.001					<0.001
Diabetic nephropathy	371 (26.2)	60 (25.9)	56 (16.4)	**487 (24.5)**	-	179 (27.5)	41 (28.5)	47 (23.4)	**267 (26.8)**	-
Glomerulonephritis	185 (13.1)	31 (13.4)	96 (28.1)	**312 (15.7)**	-	81 (12.5)	20 (13.9)	52 (25.9)	**153 (15.4)**	-
Interstitial nephritis	234 (16.5)	41 (17.7)	112 (32.7)	**387 (19.5)**	-	104 (16.0)	38 (26.4)	58 (28.9)	**200 (20.1)**	-
Multisystem	365 (25.8)	61 (26.3)	46 (13.5)	**472 (23.7)**	-	160 (24.6)	23 (16.0)	26 (12.9)	**209 (21.0)**	-
Unknown	260 (18.4)	39 (16.8)	32 (9.4)	**331 (16.6)**	-	126 (19.4)	22 (15.3)	18 (9.0)	**166 (16.7)**	-
**First KRT modality§**					<0.001					<0.001
Hemodialysis	1,268 (89.5)	69 (29.7)	203 (59.4)	**1,540 (77.3)**	-	573 (88.2)	37 (25.5)	127 (63.2)	**737 (74.0)**	**-**
Peritoneal dialysis	141 (10.0)	162 (69.8)	118 (34.5)	**421 (21.1)**	-	73 (11.2)	108 (74.5)	60 (29.9)	**241 (24.2)**	**-**
Kidney transplant	NA	NA	NA	**11 (0.6)**	-	NA	NA	NA	**11 (1.1)**	**-**

Values are n (%), mean ± SD or median [interquartile range]. Abbreviations: SIMD: Scottish index of multiple deprivation. NAs represent redacted data that might be considered potentially identifiable, including count data ≤5. §Social deprivation status (SIMD) was missing in 0.4% and 0.3%, primary kidney disease was missing in 0.1% and 0.1%, and KRT modality was missing in 1.0% and 0.7% of all patients with myocardial infarction and stroke, respectively.

Supplementary table 3. Count data and age-standardized incidence rate per 100,000 of myocardial infarction (left panel) and stroke (right panel) in men and women with kidney failure between 1996 and 2016.

Year	Myocardial infarction						
	Number of cases (both sexes), n	Estimated incidence rate *per* 100,000					
		Men			Women		
		Overall	Lower 95% CI	Upper 95% CI	Overall	Lower 95% CI	Upper 95% CI
**1996**	67	4,376	3,998	4,785	3,268	2,982	3,593
**1997**	82	4,172	3,870	4,508	3,119	2,873	3,384
**1998**	70	3,988	3,718	4,270	2,977	2,764	3,211
**1999**	68	3,823	3,585	4,078	2,855	2,658	3,064
**2000**	70	3,677	3,457	3,919	2,747	2,562	2,942
**2001**	105	3,544	3,330	3,771	2,647	2,480	2,839
**2002**	93	3,417	3,207	3,636	2,557	2,387	2,733
**2003**	90	3,303	3,104	3,511	2,466	2,307	2,638
**2004**	87	3,190	3,002	3,391	2,384	2,232	2,548
**2005**	88	3,090	2,910	3,283	2,310	2,162	2,466
**2006**	100	3,003	2,827	3,189	2,244	2,104	2,399
**2007**	101	2,919	2,752	3,105	2,180	2,042	2,329
**2008**	103	2,838	2,669	3,010	2,118	1,984	2,264
**2009**	113	2,745	2,589	2,914	2,051	1,918	2,188
**2010**	123	2,641	2,483	2,802	1,971	1,846	2,108
**2011**	98	2,520	2,376	2,673	1,883	1,762	2,011
**2012**	119	2,389	2,252	2,539	1,787	1,674	1,907
**2013**	110	2,251	2,118	2,390	1,681	1,573	1,797
**2014**	97	2,109	1,981	2,247	1,574	1,472	1,689
**2015**	101	1,965	1,832	2,110	1,470	1,363	1,583
**2016**	90	1,835	1,692	1,988	1,369	1,257	1,491

Year	Stroke						
	Number of cases (both sexes), n	Estimated incidence rate *per* 100,000					
		Men			Women		
		Overall	Lower 95% CI	Upper 95% CI	Overall	Lower 95% CI	Upper 95% CI
**1996**	34	1,978	1,795	2,175	2,234	2,031	2,468
**1997**	34	1,889	1,724	2,068	2,135	1,944	2,341
**1998**	48	1,803	1,653	1,970	2,039	1,862	2,233
**1999**	39	1,724	1,584	1,881	1,951	1,785	2,132
**2000**	52	1,647	1,518	1,787	1,863	1,711	2,031
**2001**	49	1,575	1,454	1,708	1,781	1,641	1,935
**2002**	46	1,505	1,391	1,629	1,702	1,571	1,846
**2003**	42	1,437	1,332	1,553	1,625	1,500	1,762
**2004**	50	1,375	1,276	1,482	1,555	1,435	1,680
**2005**	47	1,313	1,220	1,415	1,486	1,374	1,605
**2006**	54	1,255	1,164	1,352	1,419	1,314	1,534
**2007**	45	1,200	1,114	1,292	1,357	1,257	1,468
**2008**	46	1,147	1,064	1,238	1,298	1,200	1,399
**2009**	47	1,096	1,016	1,183	1,240	1,146	1,342
**2010**	56	1,047	968	1,130	1,184	1,094	1,280
**2011**	40	1,002	925	1,083	1,131	1,045	1,226
**2012**	45	958	882	1,037	1,082	999	1,174
**2013**	52	914	841	992	1,034	949	1,127
**2014**	50	875	804	951	987	906	1,080
**2015**	55	837	765	914	945	863	1,032
**2016**	52	799	729	875	903	824	990

Abbreviations: CI – confidence interval.

Supplementary table 4. Baseline characteristics of patients with kidney failure and incident myocardial infarction (left panel) and stroke (right panel) between 2009 and 2016, in 2-year groups.

	Myocardial infarction						Stroke					
	**2009 to 2010**	**2011 to 2012**	**2013 to 2014**	**2015 to 2016**	**Overall**	**P-value (test for trend)**	**2009 to 2010**	**2011 to 2012**	**2013 to 2014**	**2015 to 2016**	**Overall**	**P-value (test for trend)**
**Number of patients, n**	**238**	**220**	**212**	**193**	**863**	**-**	**104**	**86**	**105**	**110**	**405**	**-**
**Non-fatal event**	177 (74.4)	178 (80.9)	150 (70.8)	158 (81.9)	**663 (76.8)**	0.018	96 (92.3)	85 (98.8)	99 (94.3)	106 (96.4)	**386 (95.3)**	0.171
**Age, years**	66 (12)	67 (12)	67 (12)	65 (12)	**66 (12)**	0.258	69 (13)	67 (13)	66 (13)	66 (13)	**67 (13)**	0.371
**Sex**						0.329						0.124
Women	80 (33.6)	70 (31.8)	80 (37.7)	57 (29.5)	**287 (33.3)**	-	52 (50.0)	30 (34.9)	50 (47.6)	55 (50.0)	**187 (46.2)**	-
Men	158 (66.4)	150 (68.5)	132 (62.3)	136 (70.5)	**576 (66.8)**	-	52 (50.0)	56 (65.1)	55 (52.4)	55 (50.0)	**218 (53.8)**	-
**SIMD quintile§**						0.493						0.587
1 (most deprived)	63 (26.5)	60 (27.4)	63 (29.7)	51 (26.6)	**237 (27.5)**	-	24 (23.1)	21 (24.4)	23 (21.9)	30 (27.3)	**98 (24.2)**	-
2	58 (24.4)	60 (27.4)	42 (19.8)	49 (25.5)	**209 (24.3)**	-	22 (21.2)	22 (25.6)	29 (27.6)	17 (15.5)	**90 (22.2)**	-
3	44 (18.5)	50 (22.8)	51 (24.1)	41 (21.4)	**186 (21.6)**	-	20 (19.2)	19 (22.1)	18 (17.1)	25 (22.7)	**82 (20.2)**	-
4	42 (17.6)	25 (11.4)	26 (12.3)	32 (16.7)	**125 (14.5)**	-	18 (17.3)	17 (19.8)	21 (20.0)	21 (19.1)	**77 (19.0)**	-
5 (least deprived)	31 (13.0)	24 (11.0)	30 (14.2)	19 (9.9)	**104 (12.1)**	-	20 (19.2)	7 (8.1)	14 (13.3)	17 (15.5)	**58 (14.3)**	-
**Previous medical conditions**												
Heart failure	35 (14.7)	42 (19.1)	34 (16.0)	26 (13.5)	**137 (15.9)**	0.426	13 (12.5)	9 (10.5)	18 (17.1)	11 (10.0)	**51 (12.6)**	0.391

Myocardial infarction	15 (6.3)	17 (7.7)	24 (11.3)	17 (8.8)	**73 (8.5)**	0.278	NA	NA	7 (6.7)	6 (5.5)	**25 (6.2)**	0.509
Stroke	NA	NA	10 (4.7)	13 (6.7)	**37 (4.3)**	0.103	NA	NA	NA	NA	**10 (2.5)**	0.089
Previous coronary revascularization	15 (6.3)	13 (5.9)	15 (7.1)	17 (8.8)	**60 (7.0)**	0.669	8 (7.2)	NA	NA	NA	**16 (4.0)**	0.213
Chronic respiratory disease	39 (16.4)	31 (14.1)	52 (24.5)	38 (19.7)	**160 (18.5)**	0.043	17 (16.3)	25 (29.1)	17 (16.2)	15 (13.6)	**74 (18.3)**	0.037
Diabetes mellitus	64 (26.9)	80 (36.4)	71 (33.5)	83 (43.0)	**298 (34.5)**	0.014	26 (25.0)	35 (40.7)	44 (41.9)	50 (45.5)	**155 (38.3)**	0.037
Gastro-esophageal reflux disease	160 (67.2)	156 (70.9)	149 (70.3)	148 (76.7)	**613 (71.0)**	0.422	74 (71.2)	58 (67.4)	80 (76.2)	86 (78.2)	**298 (73.6)**	0.339
Gout	30 (12.6)	38 (17.3)	49 (23.1)	49 (25.4)	**166 (19.2)**	0.006	16 (15.4)	11 (12.8)	17 (16.2)	18 (16.4)	**62 (15.3)**	0.885
**Renal history**												
**Primary kidney disease§**						0.007						0.286
Diabetic nephropathy	56 (23.5)	66 (30.0)	53 (25.0)	71 (36.8)	**246 (28.5)**	-	28 (26.9)	26 (30.2)	35 (33.3)	45 (40.9)	**134 (33.1)**	-
Glomerulonephritis	45 (18.9)	29 (13.2)	27 (12.7)	35 (18.1)	**136 (15.8)**	-	11 (10.6)	11 (12.8)	14 (13.3)	16 (14.5)	**52 (12.8)**	-
Interstitial nephritis	59 (24.8)	46 (20.9)	41 (19.3)	35 (18.1)	**181 (21.0)**	-	15 (14.4)	19 (22.1)	22 (21.0)	17 (15.5)	**73 (18.0)**	-
Multisystem	47 (19.7)	43 (19.5)	50 (23.6)	38 (19.7)	**178 (20.6)**	-	32 (30.8)	16 (18.6)	19 (18.1)	17 (15.5)	**84 (20.7)**	-
Unknown	31 (13.0)	35 (15.9)	41 (19.3)	14 (7.3)	**121 (14.0)**	-	18 (17.3)	14 (16.3)	15 (14.3)	15 (13.6)	**62 (15.3)**	-
**First KRT modality§**						0.455						0.153
Hemodialysis	181 (76.1)	174 (79.1)	168 (79.2)	144 (74.6)	**667 (77.4)**	-	75 (72.1)	72 (83.7)	78 (74.3)	86 (78.2)	**311 (76.8)**	-
Peritoneal dialysis	53 (22.3)	38 (17.3)	40 (18.9)	43 (22.3)	**174 (20.2)**	-	27 (26.0)	10 (11.6)	24 (22.9)	21 (19.1)	**82 (20.2)**	-

Kidney transplant	NA	NA	NA	NA	**8 (0.9)**	-	NA	NA	NA	NA	**9 (2.2)**	-
**KRT modality at event**						0.492						0.879
Hemodialysis	167 (70.2)	168 (76.4)	161 (75.9)	138 (71.5)	**634 (73.5)**	-	72 (69.2)	58 (67.4)	74 (70.5)	71 (64.5)	**275 (67.9)**	-
Peritoneal dialysis	16 (6.7)	13 (5.9)	10 (4.7)	17 (8.8)	**56 (6.5)**	-	10 (9.6)	7 (8.1)	12 (11.4)	11 (10.0)	**40 (9.9)**	-
Kidney transplant	55 (23.1)	39 (17.7)	41 (19.3)	38 (19.7)	**173 (20.0)**	-	22 (21.2)	21 (24.4)	19 (18.1)	28 (25.5)	**90 (22.2)**	-
**Medications at time of incident event**												
Calcineurin inhibitor	37 (15.5)	34 (15.5)	38 (17.9)	39 (20.2)	**148 (17.1)**	0.540	18 (17.3)	15 (17.4)	18 (17.1)	20 (18.2)	**71 (17.5)**	0.988
Mycophenolate mofetil	26 (10.9)	14 (6.4)	28 (13.2)	22 (11.4)	**90 (10.4)**	0.096	13 (12.5)	10 (11.6)	18 (17.1)	19 (17.3)	**60 (14.8)**	0.591
Vitamin-K antagonist	29 (12.2)	25 (11.4)	27 (12.7)	22 (11.4)	**103 (11.9)**	0.931	9 (8.7)	11 (12.8)	12 (11.4)	12 (10.9)	**44 (10.9)**	0.925
Aspirin	138 (58.0)	141 (64.1)	127 (59.9)	97 (50.3)	**503 (58.3)**	0.045	58 (55.8)	50 (58.1)	55 (52.4)	55 (50.0)	**218 (53.8)**	0.391
Clopidogrel	43 (18.1)	41 (18.6)	36 (17.0)	56 (29.0)	**176 (20.4)**	0.008	18 (17.3)	15 (17.4)	29 (27.6)	24 (21.8)	**86 (21.2)**	0.258
Dual antiplatelet therapy	25 (10.5)	25 (11.4)	19 (9.0)	27 (14.0)	**96 (11.1)**	0.427	12 (11.5)	8 (9.3)	12 (11.4)	11 (10.0)	**43 (10.6)**	0.863
ACE-inhibitors/ARBs	101 (42.4)	97 (44.1)	71 (33.5)	75 (38.9)	**344 (39.9)**	0.056	36 (34.6)	48 (55.8)	41 (39.0)	51 (46.4)	**176 (43.5)**	0.085
Beta-blocker	125 (52.5)	114 (51.8)	118 (55.7)	124 (64.2)	**481 (55.7)**	0.027	44 (42.3)	50 (58.1)	58 (55.2)	64 (58.2)	**216 (53.3)**	0.313

Statin therapy	152 (63.9)	141 (64.1)	157 (74.1)	130 (67.4)	**580 (67.2)**	0.092	68 (65.4)	59 (68.6)	66 (62.9)	70 (63.6)	**263 (64.9)**	0.564
**Medications at discharge***												
Aspirin	117 (66.1)	135 (75.8)	110 (73.3)	113 (71.5)	**475 (71.6)**	0.083	47 (49.0)	32 (37.6)	15 (15.2)	28 (26.4)	**122 (31.6)**	<0.001
Clopidogrel	82 (46.3)	107 (60.1)	92 (61.3)	100 (63.3)	**381 (57.5)**	0.003	14 (14.6)	33 (38.8)	42 (42.4)	57 (53.8)	**146 (37.8)**	<0.001
Dual antiplatelet therapy	72 (40.7)	100 (56.2)	89 (59.3)	97 (61.4)	**358 (54.0)**	<0.001	8 (8.3)	14 (16.5)	7 (7.0)	16 (15.1)	**45 (11.7)**	0.231
ACE-inhibitors/ARBs	77 (43.5)	78 (43.8)	57 (38.0)	62 (39.2)	**274 (41.3)**	0.459	26 (27.1)	34 (40.0)	25 (25.3)	37 (34.9)	**122 (31.6)**	0.338
Beta-blocker	116 (65.5)	118 (66.3)	106 (70.7)	110 (69.6)	**450 (67.9)**	0.699	31 (32.3)	35 (41.2)	32 (32.3)	46 (43.4)	**144 (37.3)**	0.625
Statin therapy	115 (65.0)	125 (70.2)	114 (76.0)	110 (69.6)	**464 (70.0)**	0.068	52 (54.2)	45 (52.9)	49 (49.5)	67 (63.2)	**213 (55.2)**	0.517

Values are n (%), mean ± SD or median [interquartile range]. Abbreviations: SIMD: Scottish index of multiple deprivation. *The denominator for these percentages is derived from the number of non-fatal events in that year category. NAs represent redacted data that might be considered potentially identifiable, including count data ≤5. §Social deprivation status (SIMD) was missing in 0.4% and 0.3%, primary kidney disease was missing in 0.1% and 0.1%, and KRT modality was missing in 1.0% and 0.7% of all patients with myocardial infarction and stroke, respectively.

Supplementary table 5. Crude outcomes at 1 and 3 years for patients with kidney failure and incident myocardial infarction (left panel) and stroke (right panel) between 1996 and 2016, grouped by KRT modality at time of event.

	**Myocardial infarction**					**Stroke**				
	**Hemodialysis**	**Peritoneal dialysis**	**Kidney transplant**	**Overall**	**P-value (test for trend)**	**Hemodialysis**	**Peritoneal dialysis**	**Kidney transplant**	**Overall**	**P-value (test for trend)**
**Number of patients, n**	**1,418**	**232**	**342**	**1,992**	**-**	650	145	201	**996**	**-**
**Length of stay, days**	4.00 [1.00, 9.00]	4.00 [1.00, 8.00]	3.00 [1.00, 6.00]	**3.00 [1.00, 8.00]**	0.053	6.00 [2.00, 15.00]	9.00 [3.00, 21.00]	7.00 [2.00, 16.00]	**6.00 [2.00, 17.00]**	0.020
**Cardiovascular death**										
30 days	744 (52.5)	133 (57.3)	99 (28.9)	**976 (49.0)**	<0.001	248 (38.2)	39 (26.9)	40 (19.9)	**327 (32.8)**	<0.001
1 year	930 (65.6)	161 (69.4)	126 (36.8)	**1,217 (61.1)**	<0.001	379 (58.3)	80 (55.2)	64 (31.8)	**523 (52.5)**	<0.001
3 years	1,093 (77.1)	188 (81.0)	154 (45.0)	**1,435 (72.0)**	<0.001	465 (71.5)	96 (66.2)	84 (41.8)	**645 (64.8)**	<0.001
**All-cause death**										
30 days	746 (52.6)	133 (57.3)	100 (29.2)	**979 (49.1)**	<0.001	255 (39.2)	41 (28.3)	41 (20.4)	**337 (33.8)**	<0.001
1 year	969 (68.3)	164 (70.7)	133 (38.9)	**1,266 (63.6)**	<0.001	415 (63.8)	87 (60.0)	70 (34.8)	**572 (57.4)**	<0.001
3 years	1,167 (82.3)	196 (84.5)	168 (49.1)	**1,531 (76.9)**	<0.001	525 (80.8)	109 (75.2)	97 (48.3)	**731 (73.4)**	<0.001
**Non-fatal events***										

**Bleeding**										
1 year	50 (5.3)	7 (4.8)	11 (4.0)	**68 (5.0)**	0.902	47 (7.8)	12 (8.6)	10 (5.1)	**69 (7.3)**	0.430
3 years	98 (10.5)	13 (8.9)	25 (9.1)	**136 (10.0)**	0.709	71 (11.8)	22 (15.7)	20 (10.2)	**113 (12.0)**	0.270
**Heart failure**										
1 year	115 (12.3)	19 (13.0)	26 (9.4)	**160 (11.8)**	0.949	25 (4.1)	NA	NA	**38 (4.0)**	0.130
3 years	148 (15.8)	27 (18.5)	40 (14.5)	**215 (15.8)**	0.723	40 (6.6)	12 (8.6)	12 (6.1)	**64 (6.8)**	0.614
**Myocardial infarction**										
1 year	246 (26.3)	39 (26.7)	92 (33.3)	**377 (27.8)**	<0.001	27 (4.5)	NA	NA	**36 (3.8)**	0.196
3 years	290 (31.0)	43 (29.5)	103 (37.3)	**436 (32.1)**	<0.001	38 (6.3)	7 (5.0)	7 (3.6)	**52 (5.5)**	0.409
**Stroke**										
1 year	25 (2.7)	NA	NA	**30 (2.2)**	0.334	97 (16.1)	23 (16.4)	40 (20.3)	**160 (17.0)**	0.243
3 years	42 (4.5)	NA	NA	**55 (4.1)**	0.685	115 (19.0)	27 (19.3)	52 (26.4)	**194 (20.6)**	0.036
**Subsequent coronary revascularization**										
1 year	NA	NA	NA	**9 (0.7)**	0.519	-	-	-	**-**	-
3 years	17 (1.8)	5 (3.4)	9 (3.3)	**31 (2.3)**	0.116	-	-	-	**-**	-

Values are median [interquartile range] or n (%). *The denominator for these percentages is derived from the number of non-fatal events in that KRT category. NAs represent redacted data that might be considered potentially identifiable, including count data ≤5.

Supplementary table 6. Predicted probability of cardiovascular mortality rate at 1 year (from generalized linear models) following incident myocardial infarction (left panel) and stroke (right panel) between 1996 and 2016, grouped by sex.

Year	Myocardial infarction						Year	Stroke					
	Men			Women				Men			Women		
	Predicted probability (%)	Lower 95% CI	Upper 95% CI	Predicted probability (%)	Lower 95% CI	Upper 95% CI		Predicted probability (%)	Lower 95% CI	Upper 95% CI	Predicted probability (%)	Lower 95% CI	Upper 95% CI
**1996**	76.64	70.71	81.68	76.78	70.60	81.99	**1996**	63.47	53.89	72.09	67.55	57.98	75.85
**1997**	75.13	69.21	80.24	75.28	69.08	80.58	**1997**	62.42	53.08	70.92	66.55	57.19	74.77
**1998**	73.56	67.65	78.72	73.71	67.51	79.09	**1998**	61.36	52.24	69.75	65.55	56.38	73.69
**1999**	71.92	66.04	77.14	72.08	65.87	77.54	**1999**	60.29	51.38	68.56	64.52	55.54	72.59
**2000**	70.23	64.37	75.48	70.39	64.18	75.92	**2000**	59.21	50.49	67.38	63.49	54.68	71.48
**2001**	68.47	62.65	73.76	68.64	62.44	74.24	**2001**	58.12	49.58	66.20	62.44	53.78	70.37
**2002**	66.66	60.87	71.99	66.84	60.64	72.50	**2002**	57.02	48.63	65.02	61.38	52.86	69.25
**2003**	64.81	59.04	70.16	64.98	58.79	70.71	**2003**	55.91	47.65	63.86	60.31	51.90	68.14
**2004**	62.90	57.16	68.30	63.08	56.89	68.87	**2004**	54.80	46.64	62.71	59.22	50.91	67.04
**2005**	60.96	55.23	66.39	61.14	54.94	67.00	**2005**	53.68	45.60	61.58	58.13	49.88	65.95
**2006**	58.97	53.25	64.46	59.16	52.95	65.10	**2006**	52.56	44.53	60.47	57.03	48.82	64.87
**2007**	56.96	51.23	62.51	57.15	50.91	63.18	**2007**	51.44	43.42	59.39	55.93	47.73	63.82
**2008**	54.93	49.18	60.55	55.12	48.85	61.24	**2008**	50.32	42.29	58.33	54.82	46.60	62.78
**2009**	52.88	47.09	58.59	53.07	46.76	59.29	**2009**	49.19	41.12	57.30	53.70	45.44	61.76
**2010**	50.82	44.99	56.63	51.02	44.65	57.34	**2010**	48.07	39.94	56.30	52.58	44.25	60.77
**2011**	48.76	42.87	54.68	48.95	42.54	55.40	**2011**	46.95	38.73	55.33	51.46	43.03	59.80
**2012**	46.70	40.75	52.75	46.89	40.42	53.47	**2012**	45.83	37.51	54.39	50.33	41.79	58.86
**2013**	44.65	38.63	50.83	44.84	38.32	51.55	**2013**	44.71	36.27	53.47	49.21	40.53	57.94
**2014**	42.62	36.54	48.94	42.81	36.23	49.66	**2014**	43.60	35.03	52.58	48.08	39.25	57.04
**2015**	40.62	34.47	47.08	40.81	34.18	47.78	**2015**	42.50	33.78	51.72	46.96	37.96	56.16
**2016**	38.64	32.44	45.24	38.83	32.17	45.94	**2016**	41.41	32.53	50.87	45.84	36.67	55.31

Supplementary figure 1. Schematic illustrating the linkage of national datasets to create the final study cohort and to define the study outcomes. *Abbreviations: KRT – kidney replacement therapy.*

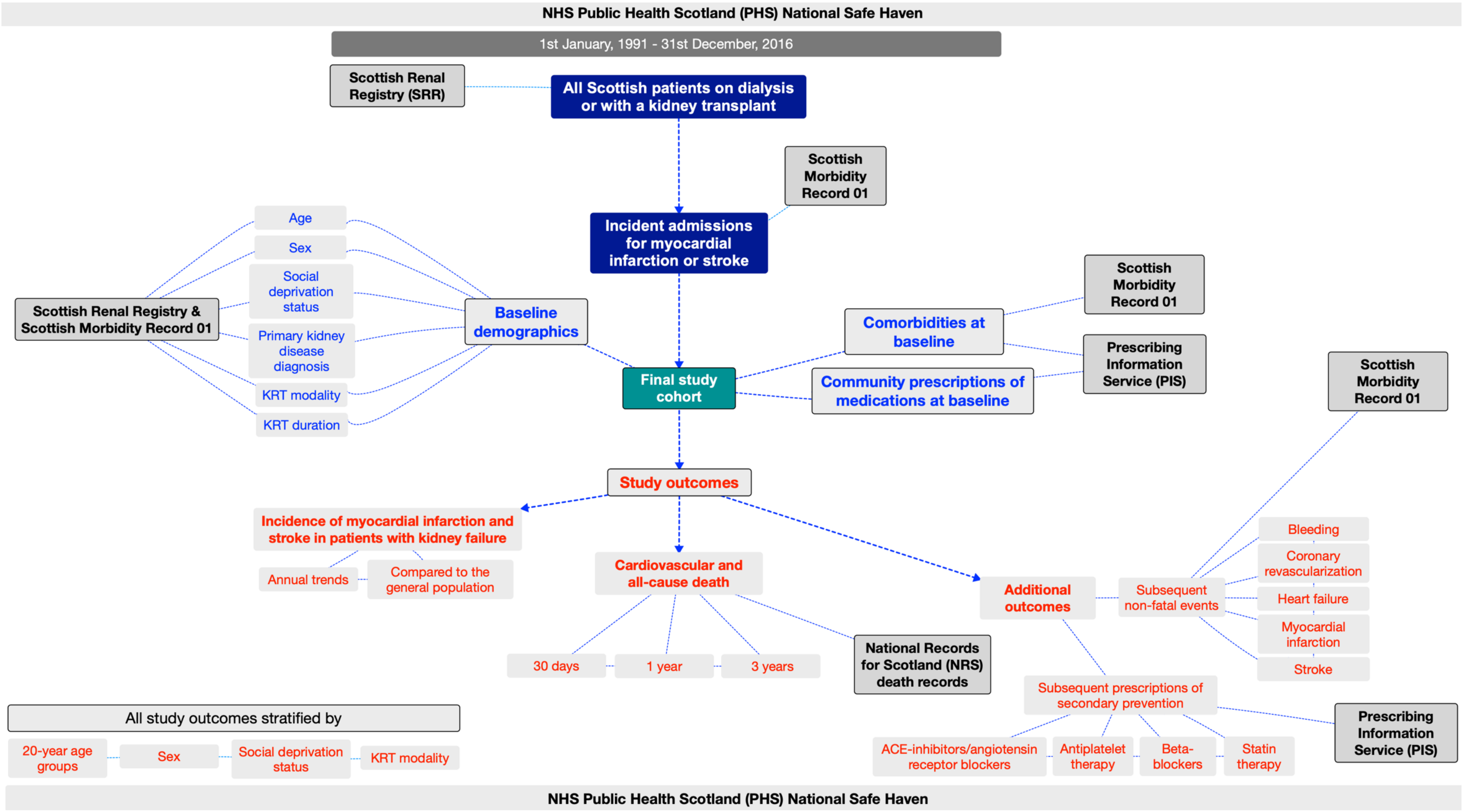

Supplementary figure 2. Stack plot illustrating the underlying etiology of kidney disease for patients with incident myocardial infarction (left panel) and stroke (right panel) according to year of event.

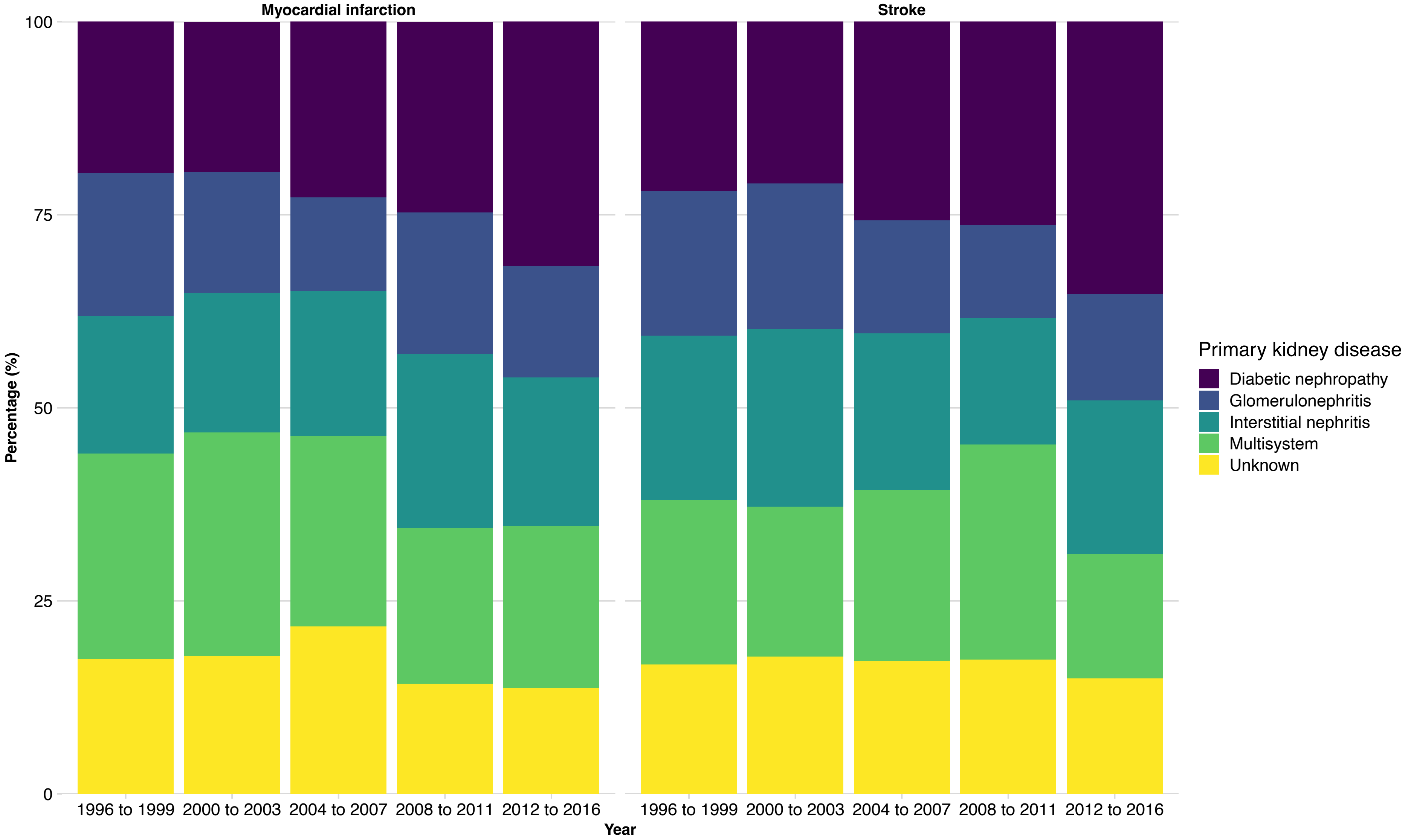

Supplementary figure 3. Stack plot illustrating the modality of kidney replacement therapy at time of event for patients with incident myocardial infarction (left panel) and stroke (right panel) according to year of event.

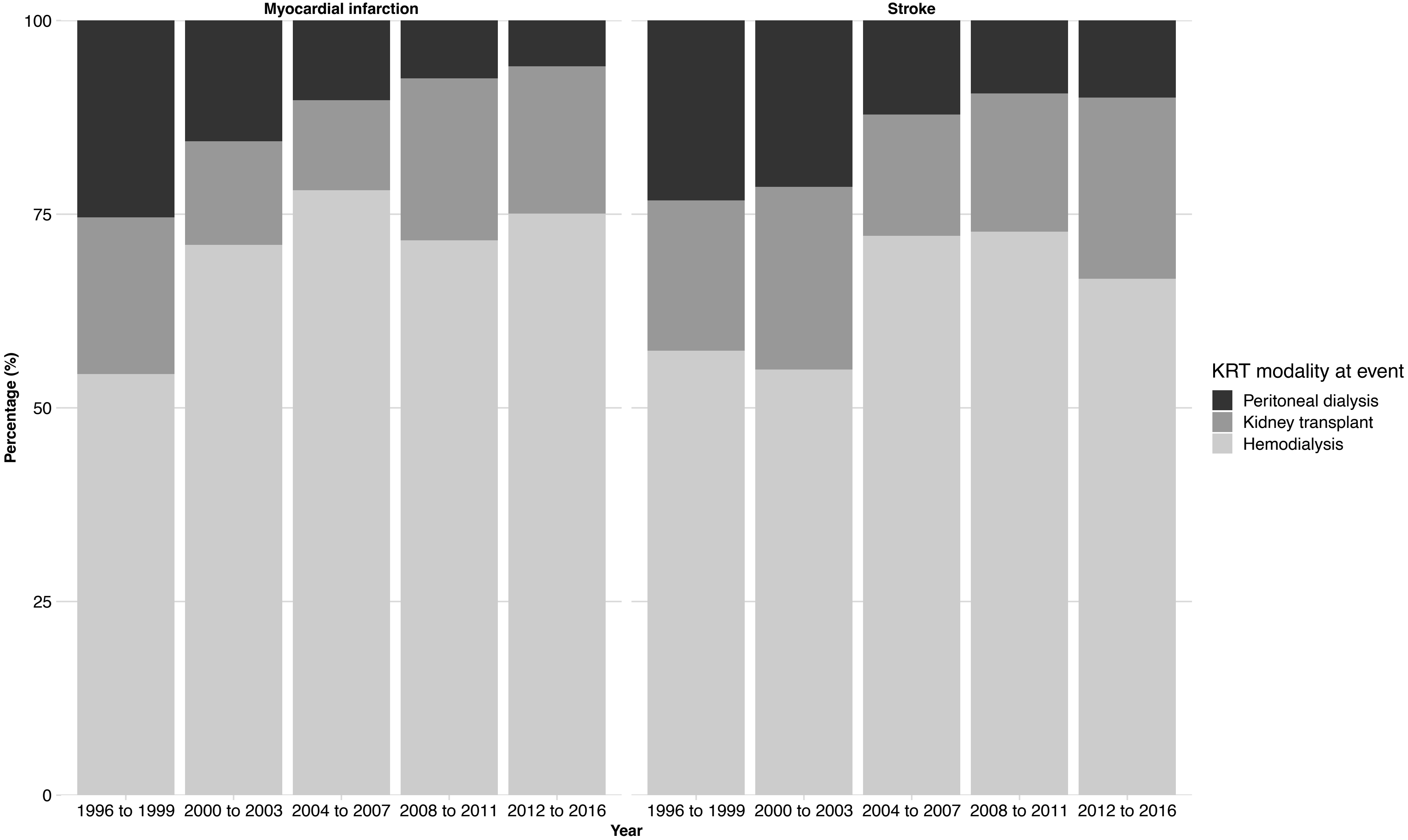

Supplementary figure 4a. Incidence of myocardial infarction in patients with kidney failure between 1996 and 2016, according to age.

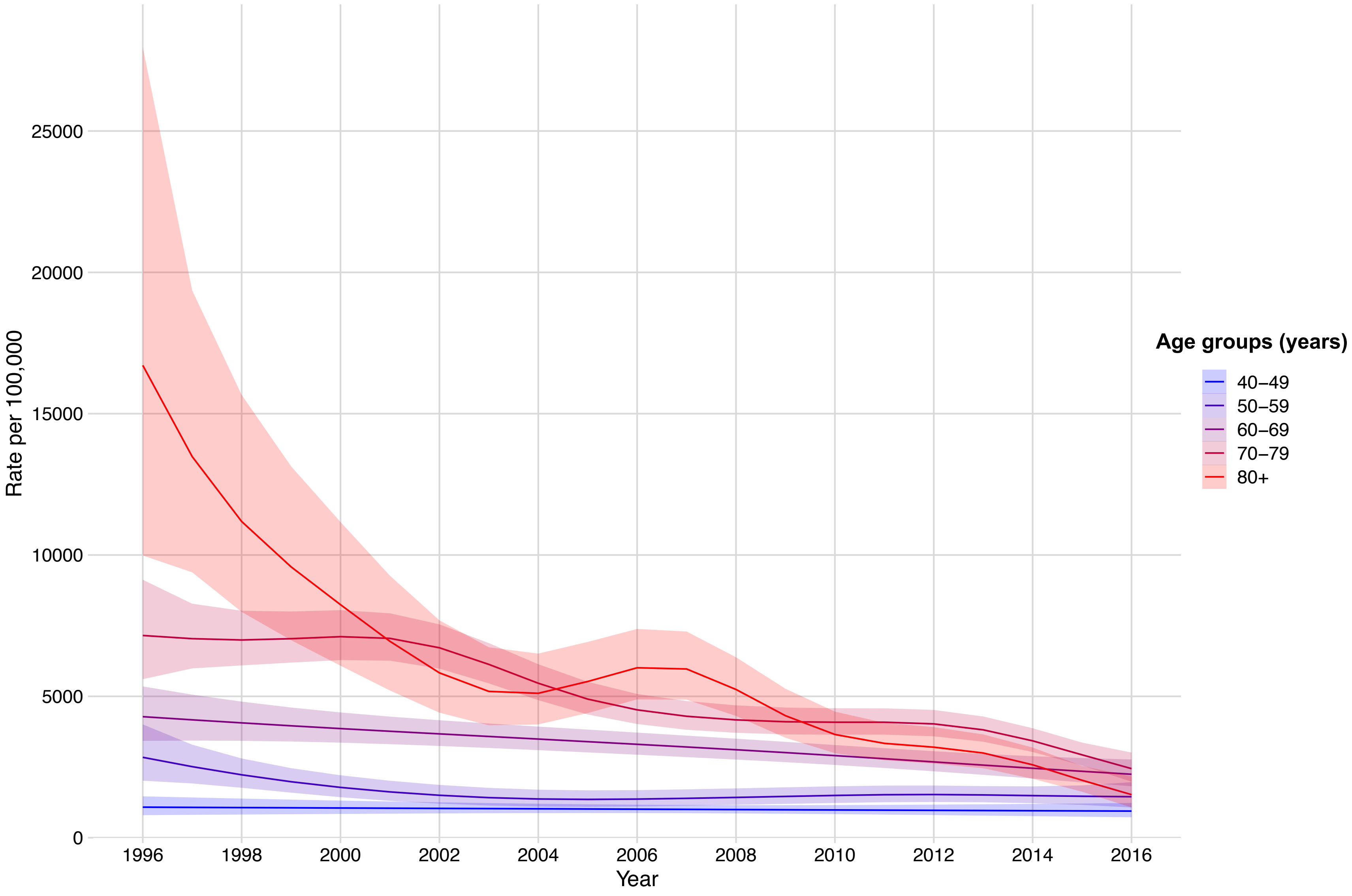

Supplementary figure 4b. Incidence of stroke in patients with kidney failure between 1996 and 2016, according to age.

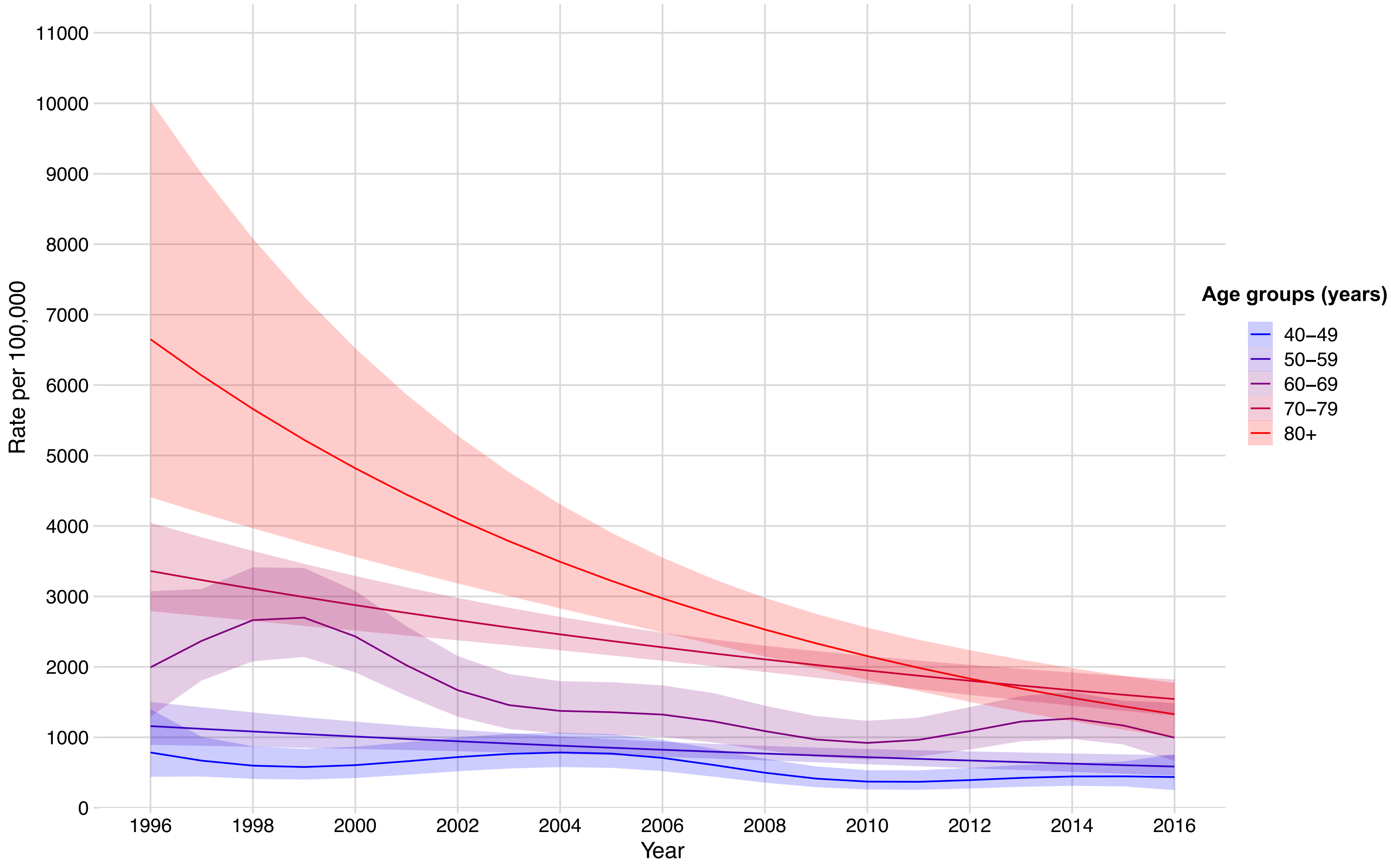

Supplementary Figure 5a. Incidence of myocardial infarction in patients with kidney failure between 1996 and 2016, according to KRT modality (i.e., hemodialysis, peritoneal dialysis, and kidney transplant) at the time of incident event.

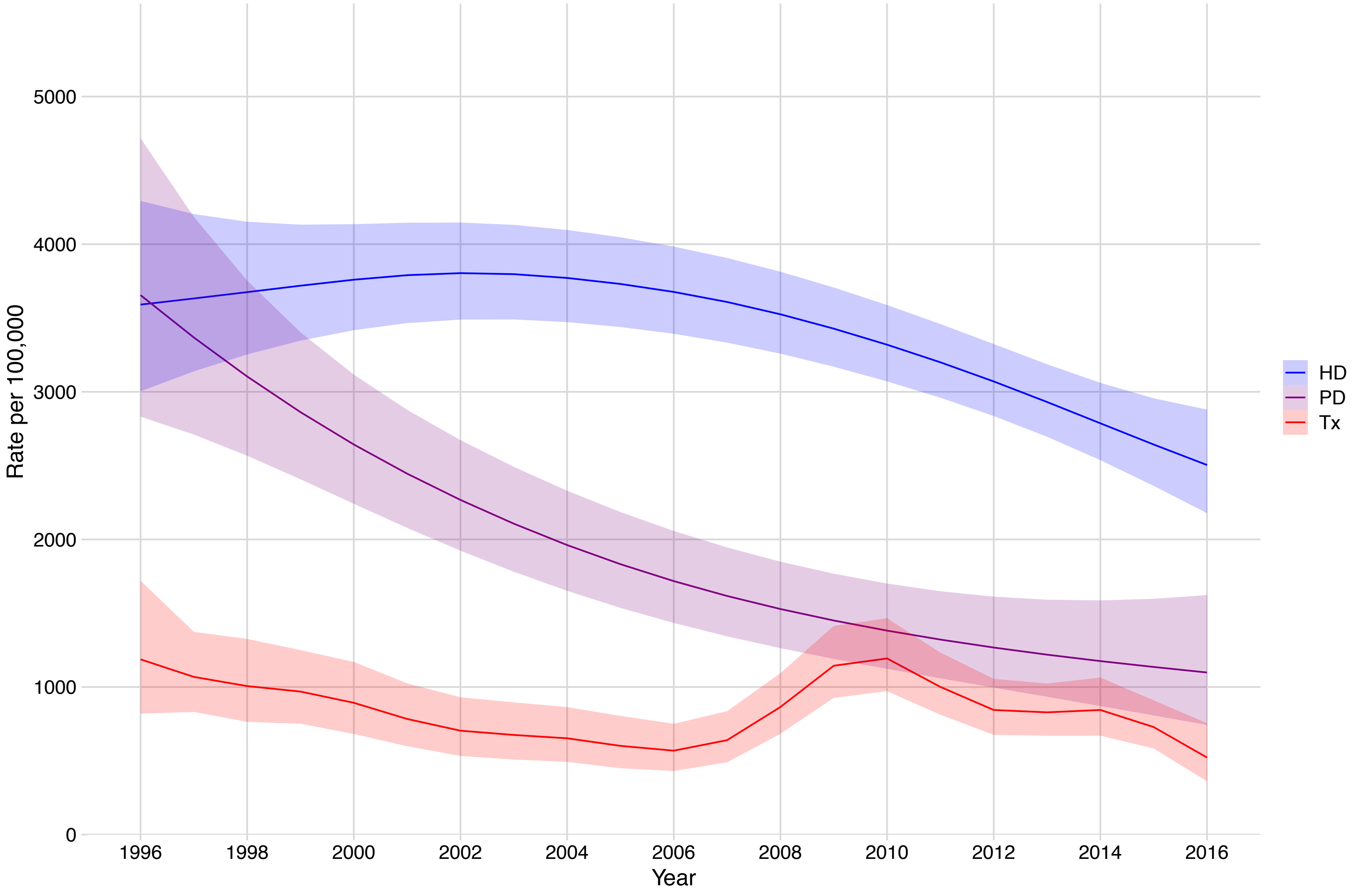

Supplementary figure 5b. Incidence of stroke in patients with kidney failure between 1996 and 2016, according to KRT modality (i.e., hemodialysis, peritoneal dialysis, and kidney transplant) at the time of incident event.

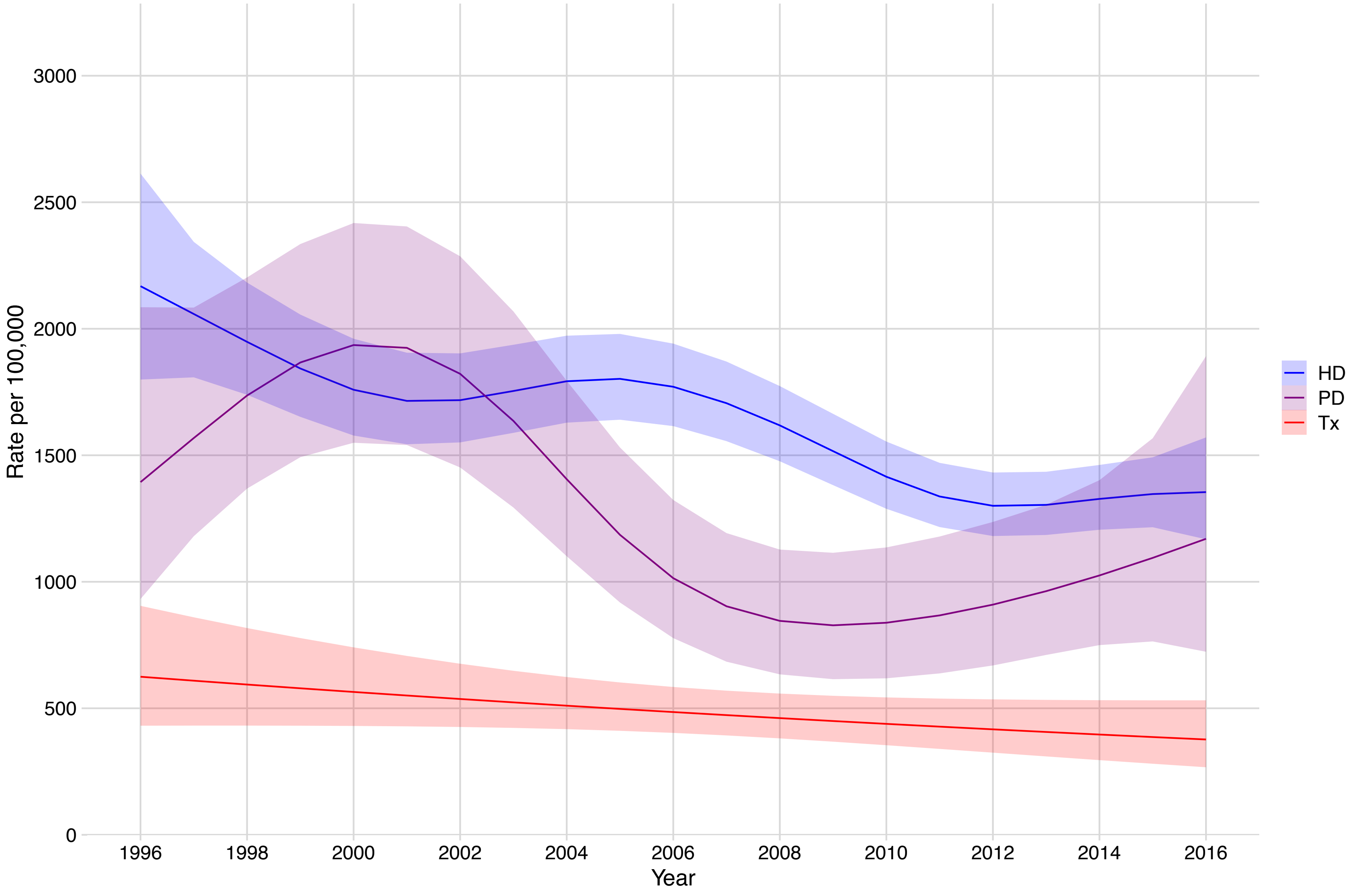

Supplementary figure 6a. IRRs for myocardial infarction for patients aged 40-69 (left panel) and ≥70 (right panel) years old between 1996 and 2014.

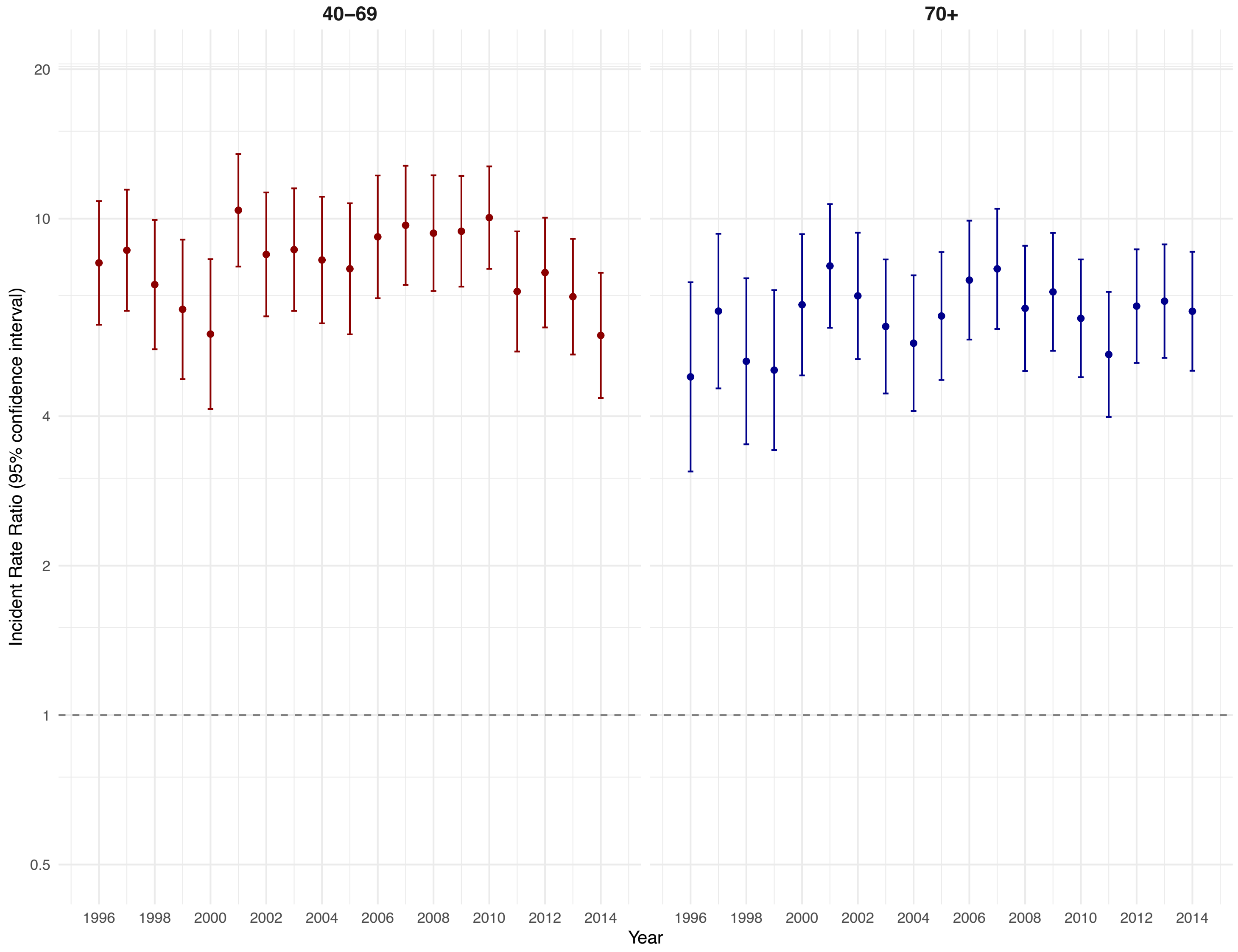

Supplementary figure 6b. IRRs for stroke for patients aged 40-69 (left panel) and ≥70 (right panel) years old between 1996 and 2014.

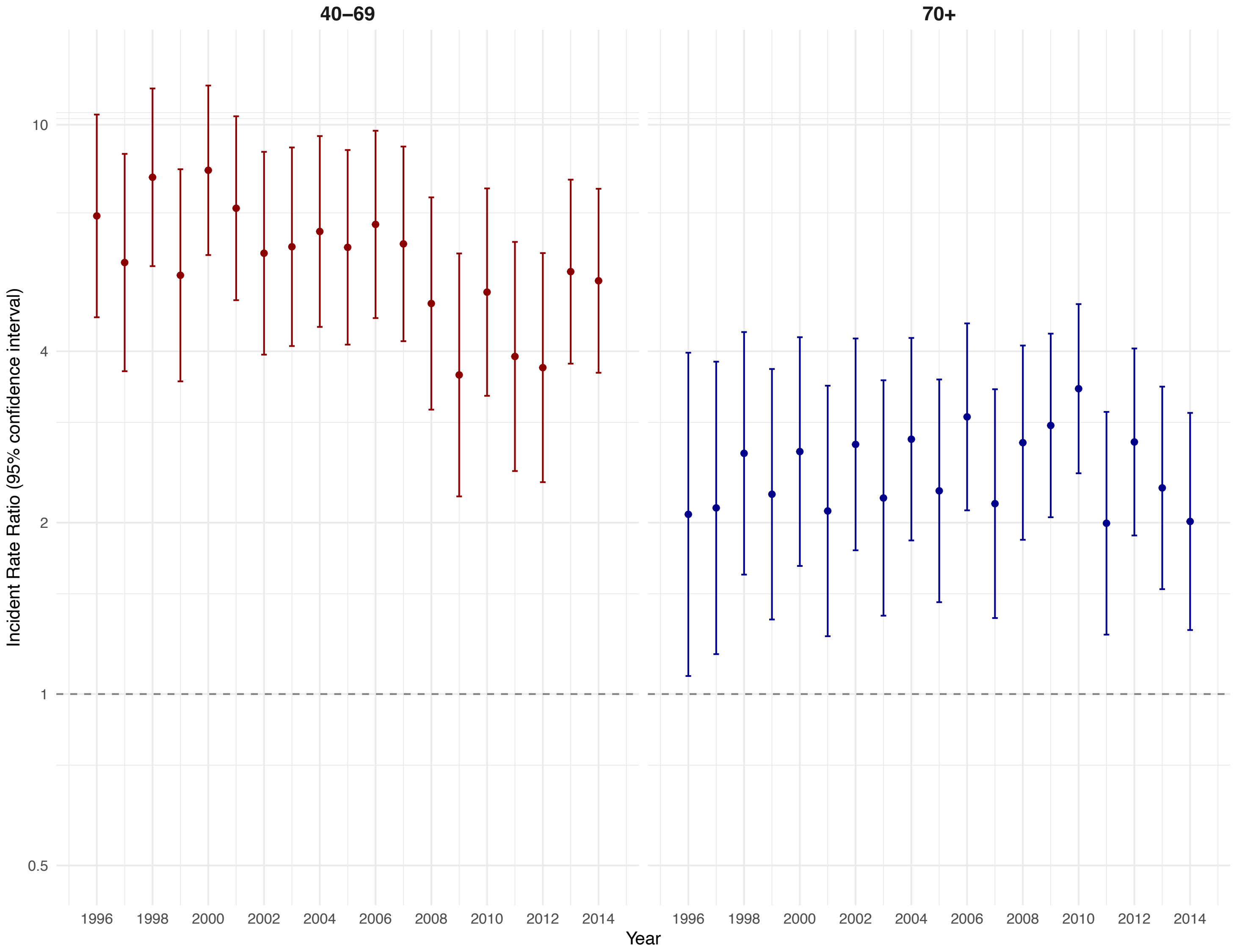

Supplementary figure 7a. IRRs for myocardial infarction between 1996 and 2014 according to KRT modality at time of incident event.

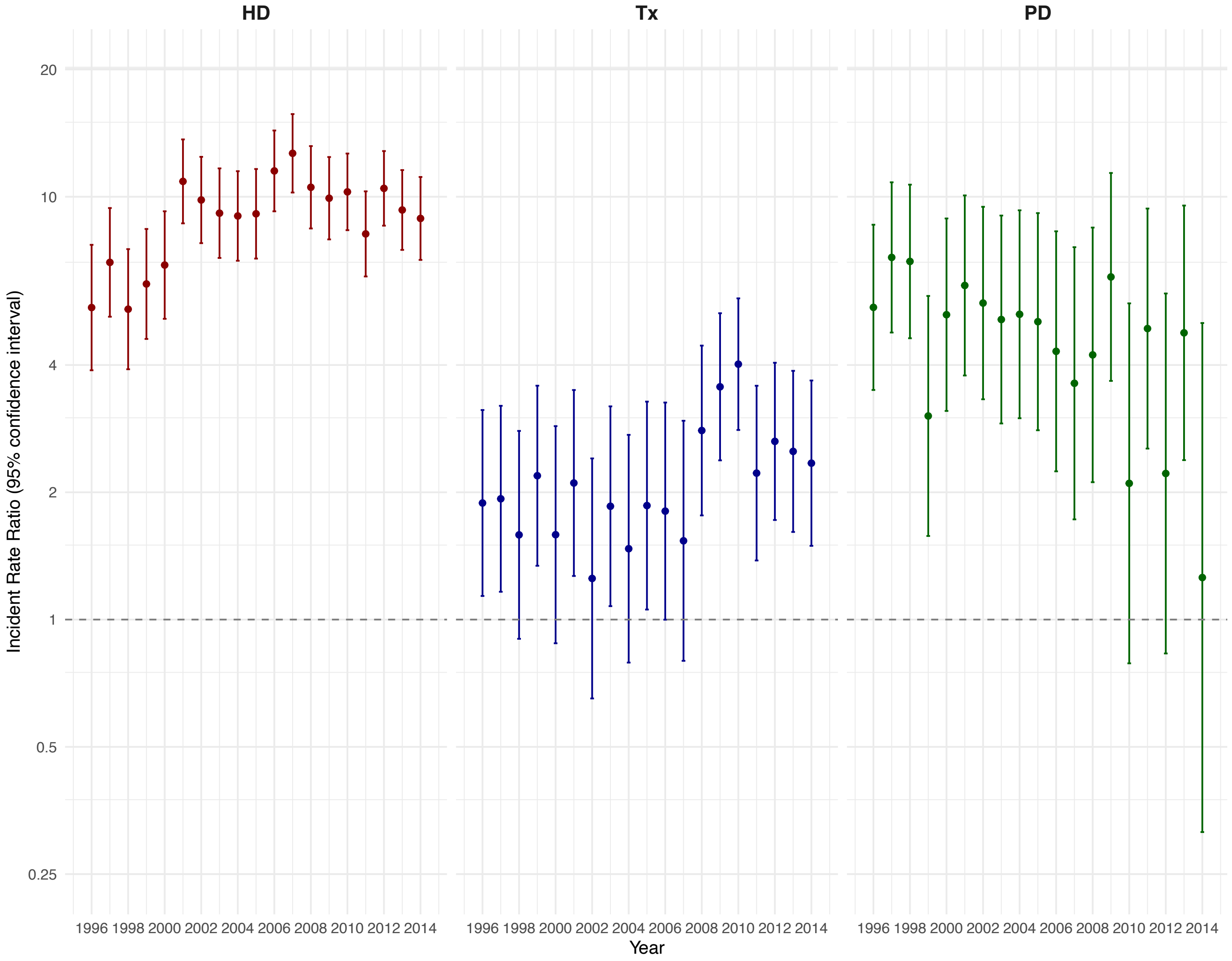

Supplementary figure 7b. IRRs for stroke between 1996 and 2014 according to KRT modality at the time of incident event.

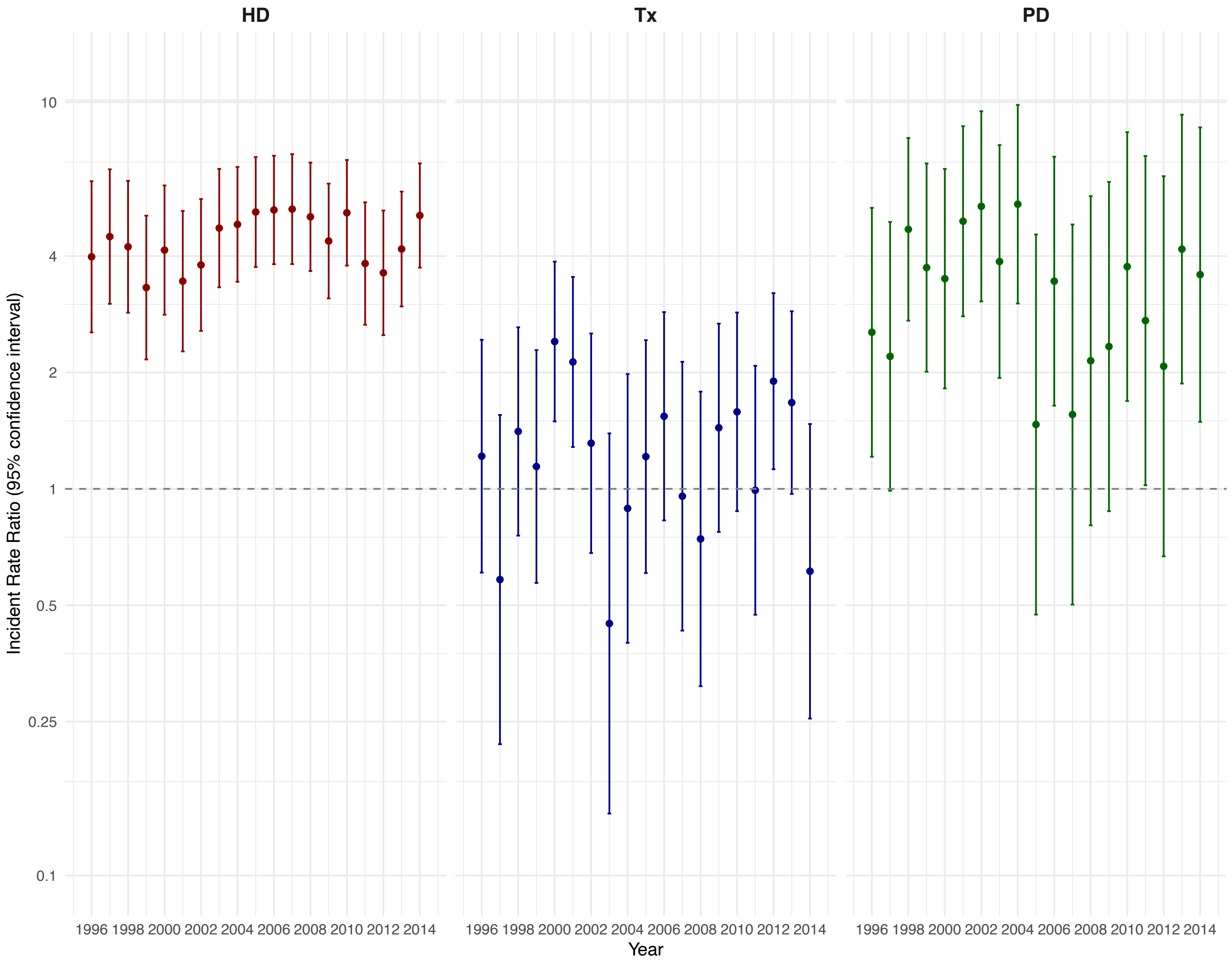

Supplementary figure 8a. Trends in the prescription of cardiovascular preventative therapies before (top panel) and after (bottom panel) incident myocardial infarction between 2009 and 2016.

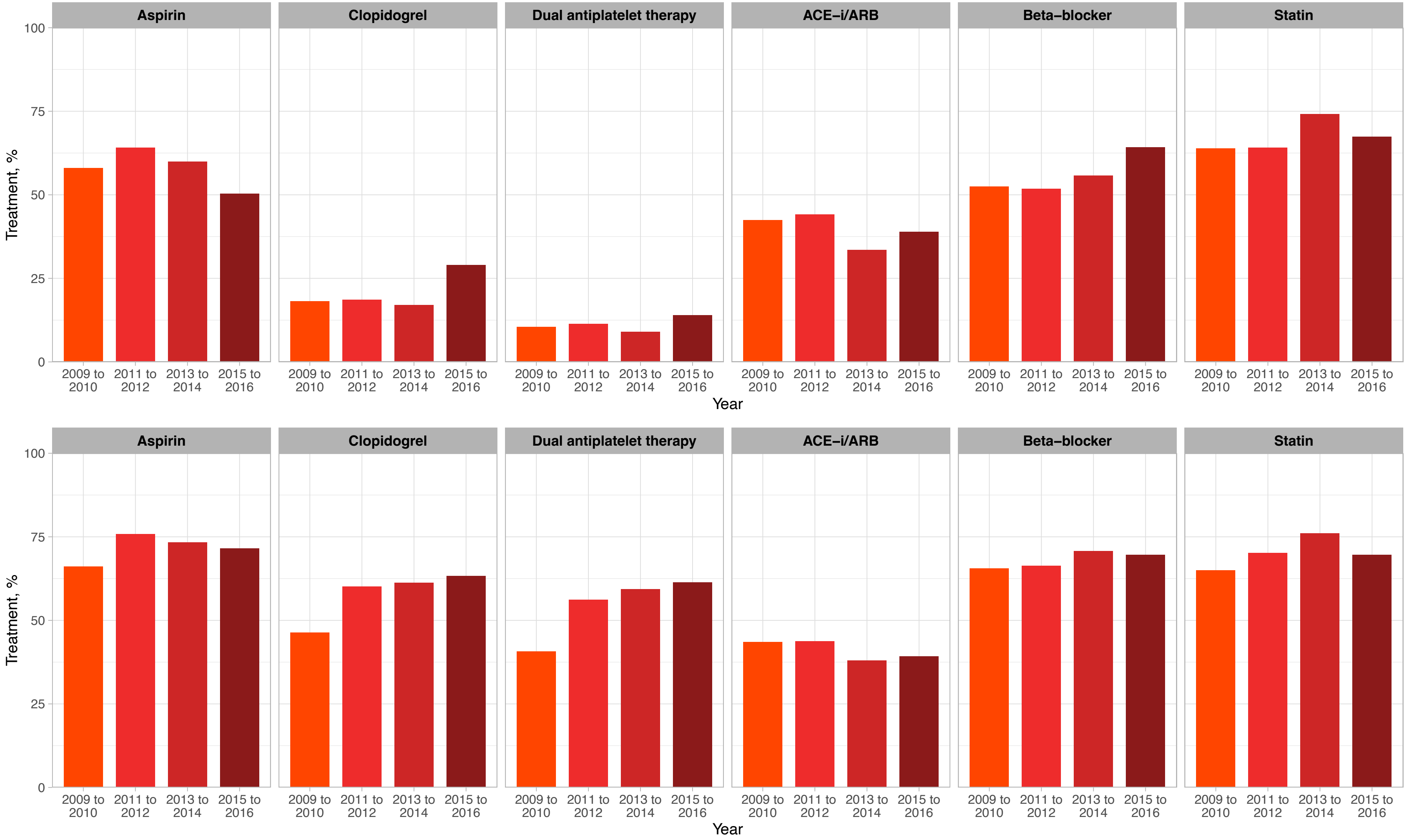

Supplementary figure 8b. Trends in the prescription of preventative therapies before (top panel) and after (bottom panel) incident stroke between 2009 and 2016.

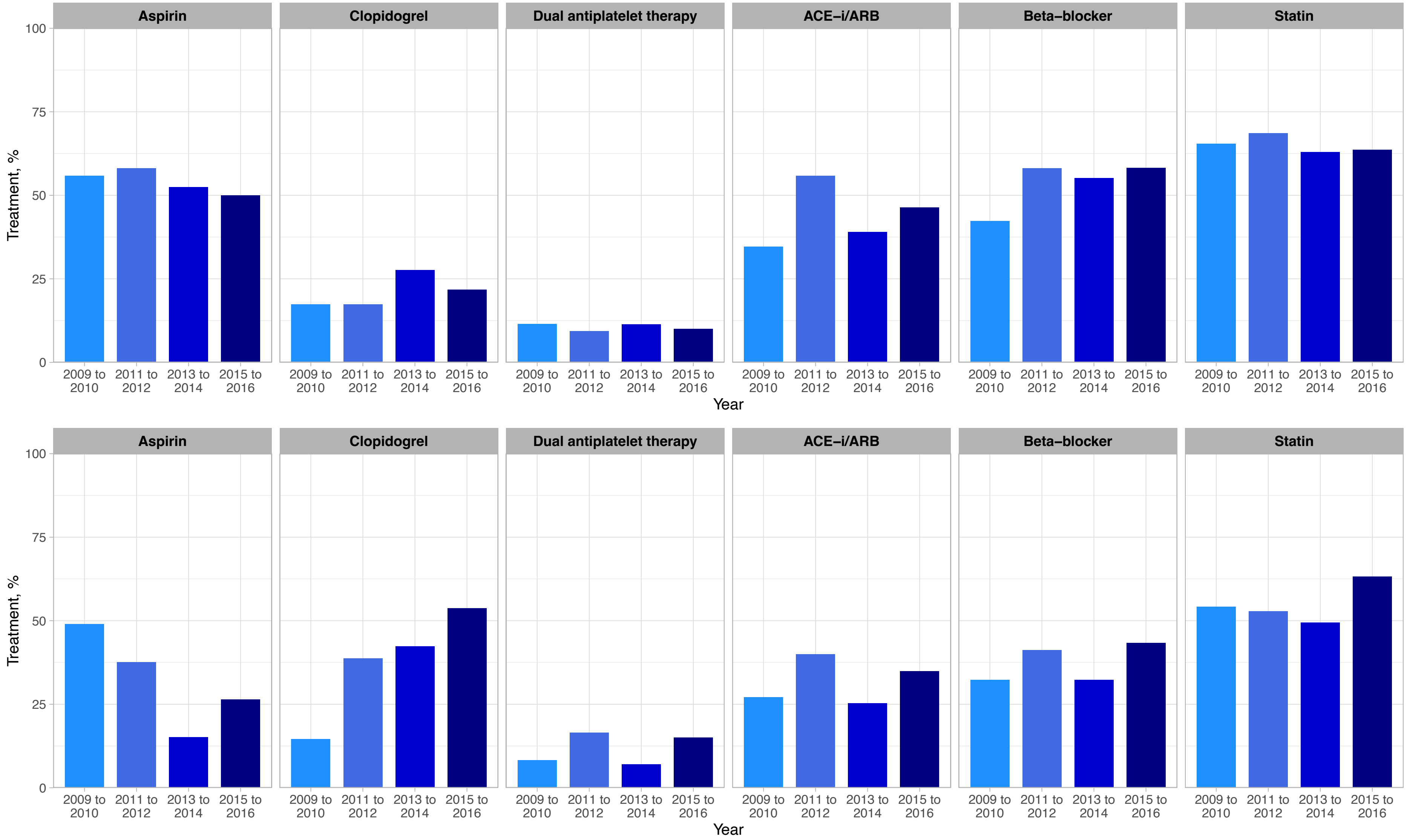

Supplementary figure 9a. Predicted probability of cardiovascular mortality at 1 year following incident myocardial infarction with kidney failure between 1996 and 2016, according to KRT modality (i.e., hemodialysis, peritoneal dialysis, kidney transplant).

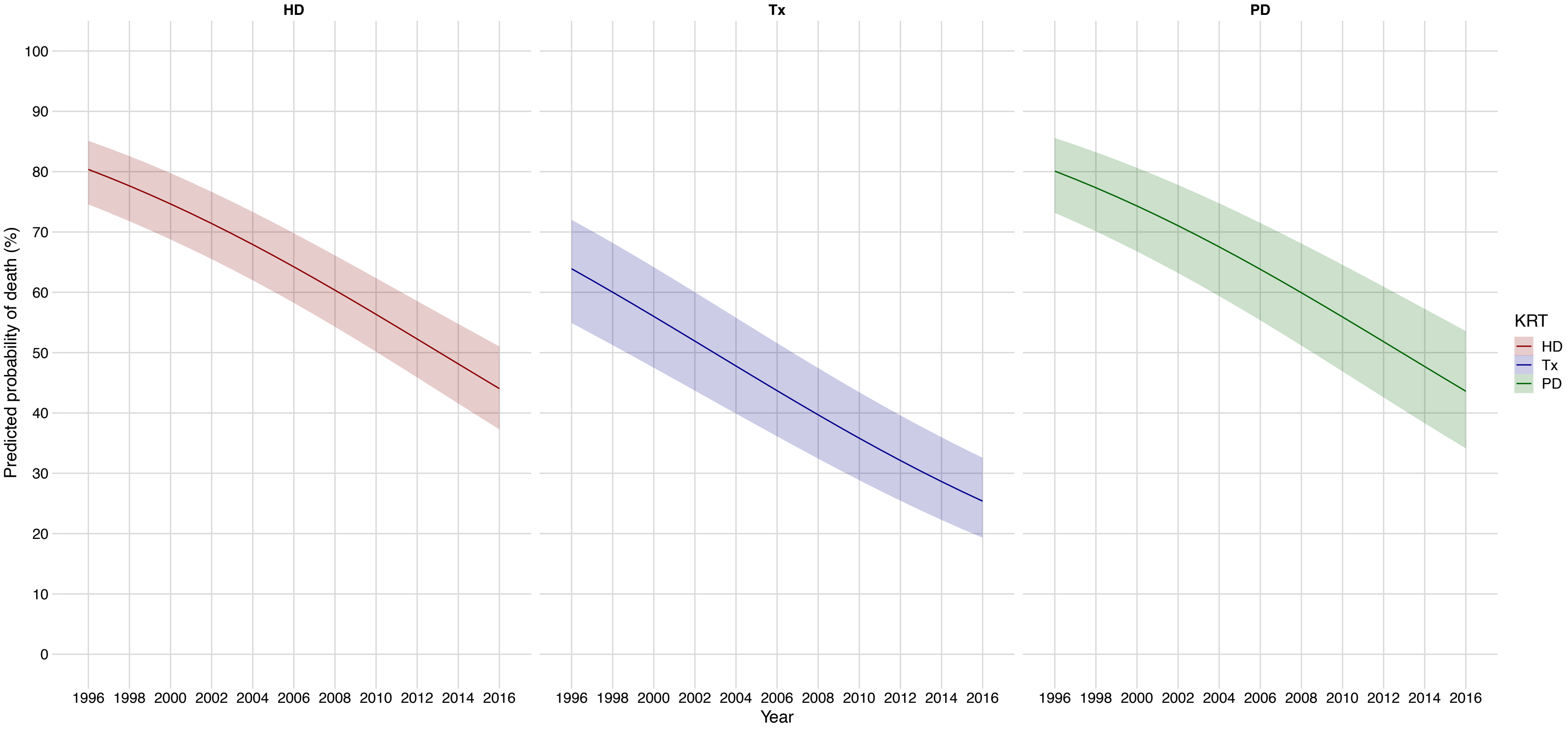

Supplementary figure 9b. Predicted probability of cardiovascular mortality at 1 year following incident stroke with kidney failure between 1996 and 2016, according to KRT modality (i.e., hemodialysis, peritoneal dialysis, kidney transplant).

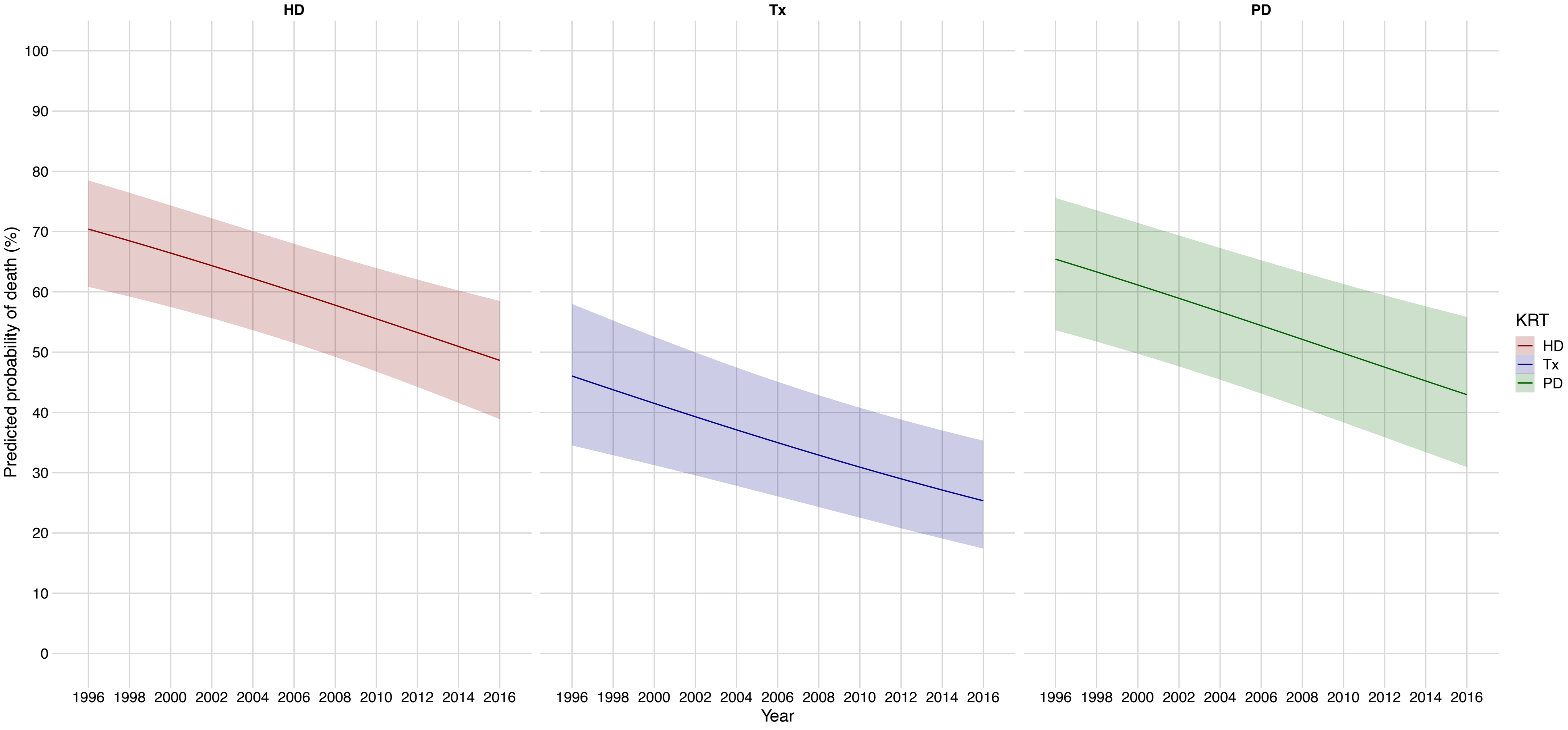

Supplementary figure 10a. Predicted probability of all-cause mortality at 1 year following incident myocardial infarction with kidney failure between 1996 and 2016, according to KRT modality (i.e., hemodialysis, peritoneal dialysis, kidney transplant).

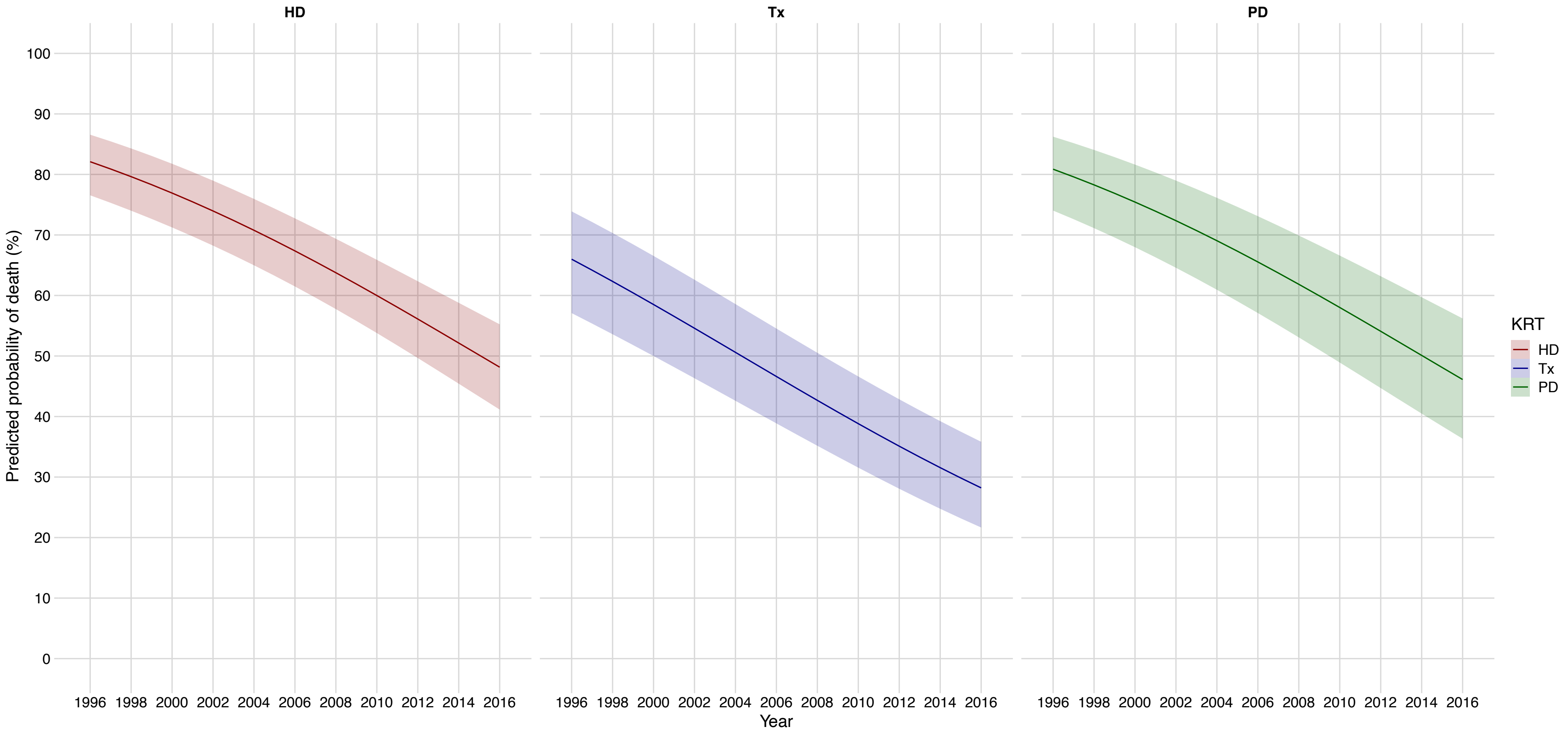

Supplementary figure 10b. Predicted probability of all-cause mortality at 1 year following incident stroke with kidney failure between 1996 and 2016, according to KRT modality (i.e., hemodialysis, peritoneal dialysis, kidney transplant).

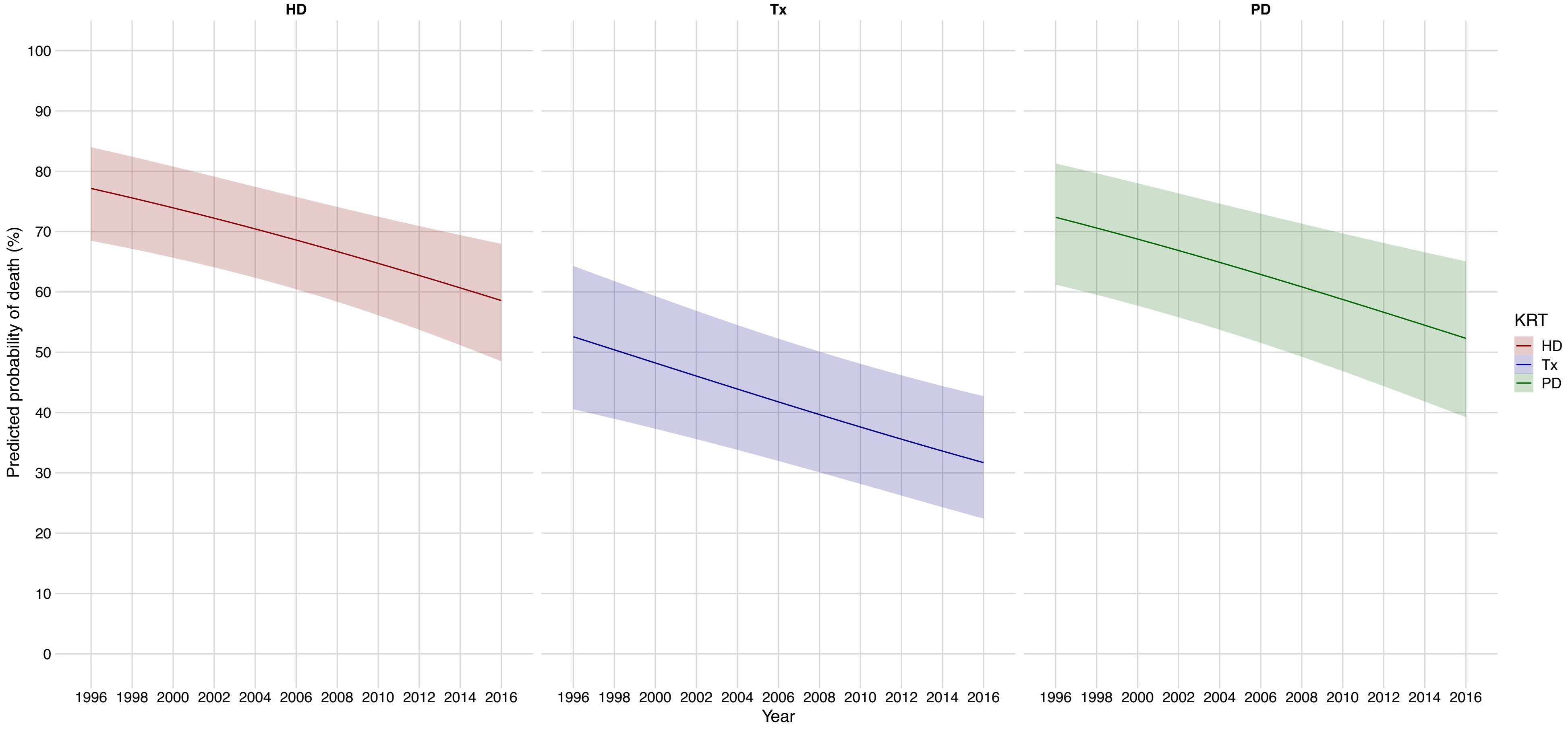

References

1. Scotland NPH. Data Support and Monitoring: SMR Completeness Estimates. 2021;2021.

2. Scotland NNS. NHS National Services Scotland (NSS) Information and Intelligence assessment of SMR01 data 2014-2015. 2015;2021.

3. Scotland NRo. National Records of Scotland Death Registry. 2021.

4. Alvarez-Madrazo S, McTaggart S, Nangle C, Nicholson E and Bennie M. Data resource profile: the Scottish national prescribing information system (PIS). *Int J Epidemiol.* 2016;45:714.
